# AKNA serves as a prognostic biomarker and restrains endometrial cancer progression via STING activation and macrophage reprogramming

**DOI:** 10.3389/fimmu.2026.1905624

**Published:** 2026-10-05

**Authors:** Yuan Tu, Lingya Xu, Chengfan Tian, Yuanling Shi, Jiaxin Yu, Chunxia Gong, Tianyu Peng, HangKun Yu, Rui Yuan, Peng Jiang

**Affiliations:** 1 Chongqing Key Laboratory of Maternal and Fetal Medicine, Department of Obstetrics and Gynecology, The First Affiliated Hospital of Chongqing Medical University, Chongqing, China; 2 Department of Gynecology, Chongqing University Qianjiang Hospital (Qianjiang Central Hospital of Chongqing), Chongqing, China; 3 International Medical College, Chongqing Medical University, Chongqing, China; 4 Department of Obstetrics and Gynecology, Women and Children’s Hospital of Chongqing Medical University, Chongqing, China

**Keywords:** AKNA, chemosensitivity, endometrial cancer, macrophage polarization, prognostic biomarker, STING

## Abstract

**Background:**

AKNA, an AT-hook transcription factor, plays a critical role in cancer development. However, its prognostic value and biological function in endometrial cancer (EC) remain unclear.

**Methods:**

To systematically investigate AKNA in EC, we integrated multi-omics data (The Cancer Genome Atlas (TCGA), Genotype-Tissue Expression (GTEx), and single-cell transcriptomics), dual independent clinical cohorts (TCGA-UCEC dataset and two institutional cohorts), and *in vitro*/*in vivo* experimental models. Immunohistochemistry, RT-qPCR and Western blotting were used for expression validation. Functional assays, including lentiviral-mediated AKNA knockdown/overexpression, CCK-8, scratch-wound, Transwell, flow cytometry, and colony formation assays were conducted to assess the effects of AKNA modulation on proliferation, migration, invasion, epithelial-mesenchymal transition, chemosensitivity and macrophage polarization. Mechanistically, chromatin immunoprecipitation (ChIP) sequencing (ChIP-seq), ChIP-qPCR, and dual-luciferase reporter assays were employed to define the transcriptional regulation of stimulator of interferon genes (STING) by AKNA. A prognostic nomogram integrating AKNA expression with clinicopathological features was developed and externally validated in independent cohorts.

**Results:**

AKNA was significantly downregulated in EC tissues compared with normal endometrium, which was associated with advanced International Federation of Gynecology and Obstetrics stage, aggressive histological subtypes, and poor prognosis. AKNA knockdown promoted EC cell proliferation, migration, invasion, and epithelial-mesenchymal transition, while enhancing M2 macrophage polarization and reducing sensitivity to paclitaxel and potentially compromising anti-tumor innate immunity. Conversely, AKNA overexpression or STING activation by cyclic GMP–AMP reversed these malignant phenotypes. Mechanistically, AKNA directly bound to the STING promoter and transcriptionally activated STING, thereby inducing TANK-binding kinase 1 and interferon regulatory factor 3 phosphorylation. The nomogram combining AKNA with clinicopathological features demonstrated high accuracy in predicting EC recurrence and outperformed single-variable models in both training and validation cohorts.

**Conclusions:**

AKNA deficiency promotes EC progression by transcriptionally suppressing STING and driving M2 macrophage polarization. AKNA serves as a robust prognostic biomarker and predictive indicator for chemotherapy and innate immune modulation, offering a promising therapeutic target for precision treatment of EC.

## 1 Introduction

Endometrial cancer (EC) is the most common gynecological malignancy in developed countries, with incidence rising substantially over recent decades. The 2022 Global Cancer Statistics reported approximately 420,242 new cases and 97,704 deaths worldwide annually (1). Most patients are diagnosed at an early stage and have a favorable prognosis (2). However, approximately 15% of patients present with advanced disease. Five-year overall survival (OS) rates vary markedly by stage, from 40-65% for International Federation of Gynecology and Obstetrics (FIGO) stage III to only 15-17% for stage IV. Treatment options for advanced or recurrent EC remain limited (3). Although immunotherapy benefits a subset of patients, objective response rates to checkpoint inhibitors are generally modest (4, 5). Therefore, novel biomarkers are urgently needed to improve prognostic stratification and guide therapeutic decisions in EC.

AKNA, a member of the AT-hook transcription factor family, has been reported to regulate diverse biological processes, including transcriptional regulation, immune response and cancer pathogenesis (6). Emerging evidence suggests that AKNA plays critical roles in multiple malignancies. In cervical cancer, AKNA single-nucleotide polymorphisms have been linked to cancer susceptibility, and human papillomavirus E6 downregulates AKNA expression to promote tumor progression (7, 8). In epithelial ovarian cancer, AKNA genetic variants confer susceptibility (9). Camargo et al. demonstrated that AKNA regulates epithelial-mesenchymal transition (EMT) during neurogenesis (10). In gastric cancer, AKNA acts as a tumor suppressor, likely by modulating EMT-associated signaling pathways (11). Collectively, these findings support a tumor-suppressive role for AKNA, and targeting AKNA may represent a promising strategy. Nevertheless, the prognostic significance and molecular mechanisms of AKNA in EC remain elusive.

This study aims to explore the prognostic significance and functional role of AKNA in EC. Our results indicate that AKNA functions as both a prognostic indicator and a tumor suppressor. Through activation of the stimulator of interferon genes (STING) pathway, AKNA promotes macrophage polarization toward an anti-tumor phenotype, thereby suppressing tumor progression. Furthermore, we established a nomogram integrating AKNA expression with clinicopathological features. This model identifies high-risk EC patients and facilitates personalized therapeutic decision-making.

## 2 Materials and methods

### 2.1 Data resources and bioinformatics analysis of AKNA

Transcriptomic and clinical data were retrieved from The Cancer Genome Atlas Uterine Corpus Endometrial Carcinoma (TCGA-UCEC) cohort, supplemented with normal controls (35 normal samples from TCGA and 142 normal endometrial samples from GTEx v8). Differential expression of AKNA between tumor and normal tissues was evaluated using the Wilcoxon rank-sum test. The Kruskal-Wallis test was performed to assess AKNA expression stratified across FIGO stages and histological grades. Associations between AKNA expression and molecular subtypes were analyzed using TISIDB (12). Protein expression data from the Clinical Proteomic Tumor Analysis Consortium were retrieved via the UALCAN database (13). Bioinformatics resources are summarized in **Supplementary Table 1**.

### 2.2 Analysis of survival outcomes, functional enrichment, and immune infiltration

Survival outcomes were analyzed using Kaplan-Meier method for both OS and recurrence-free survival (RFS). Patients were stratified according to an optimal AKNA expression cutoff determined in the TCGA-UCEC cohort, and intergroup differences were assessed using the log-rank test (14). Protein-protein interaction networks were generated using GeneMANIA and STRING (15, 16). Genes co-expressed with AKNA, along with their Pearson correlation coefficients, were retrieved from the cBioPortal co-expression module (17). Functional annotation was performed using Gene Set Enrichment Analysis (GSEA), with significance thresholds of *P* < 0.05 and false discovery rate q < 0.25. Subsequently, Gene Ontology (GO) and Kyoto Encyclopedia of Genes and Genomes (KEGG) pathway enrichment analyses were conducted using Metascape (18).

To characterize the tumor immune microenvironment, the ESTIMATE algorithm was applied to TCGA-UCEC transcriptomic data to calculate immune and stromal scores, as well as ESTIMATE scores. Tumor purity was calculated using the established trigonometric function: purity = cos (0.6049872018 + 0.000146788 × ESTIMATE score) (19). The Microenvironment Cell Populations-counter (MCP-counter) algorithm was further employed to quantify immune cell infiltration in EC tissues (20). These metrics were subsequently compared between AKNA-high and AKNA-low subgroups. Additionally, the TISIDB portal was used to explore correlations between AKNA expression and immunomodulators, including major histocompatibility complex (MHC) molecules, immunostimulators, and immunoinhibitors (12).

### 2.3 Clinical sample collection

A total of 680 patients with stages I-III EC were enrolled between December 2017 and December 2022. The training cohort (n = 395) consisted of patients from the First Affiliated Hospital of Chongqing Medical University (FAHCQMU), and the validation cohort (n = 285) was obtained from the Women and Children’s Hospital of Chongqing Medical University (WCHCQMU). All patients underwent primary surgical treatment, and EC diagnosis was confirmed by postoperative pathological examination. Clinical data, including age, body mass index, serum CA125 level, FIGO stage, histotype, lymphovascular space invasion (LVSI), p53 status, cervical stromal invasion, myometrial invasion, records of adjuvant therapy and follow-up were collected. Postoperative tissue specimens were obtained from the pathology center and processed into paraffin sections for immunohistochemical (IHC) analysis. Additionally, 20 pairs of fresh EC and adjacent normal endometrial tissues were collected for subsequent analyses. Postoperative adjuvant therapy protocols, follow-up procedures, and recurrence definitions were as previously described (21). Written informed consent was obtained from all patients for the use of their endometrial carcinoma tissue specimens in research, and the study was carried out in strict accordance with the Declaration of Helsinki. This study was approved by the ethics committees of FAHCQMU (2025–283–01) and WCHCQMU (2023–002).

### 2.4 Assessment of immunotherapy response and chemotherapy response

Immunotherapy response prediction was assessed using the Tumor Immune Dysfunction and Exclusion (TIDE) algorithm, which quantifies immune evasion through cytotoxic T lymphocyte dysfunction and exclusion. Elevated TIDE scores indicate resistance to immune checkpoint blockade. The association between AKNA expression and chemosensitivity was evaluated using the Genomics of Drug Sensitivity in Cancer (GDSC) database. Spearman correlation analysis was performed to assess the correlation between AKNA expression and drug sensitivity (half-maximal inhibitory concentration (IC50) values) or immunotherapy response (22).

### 2.5 IHC analysis

IHC was performed as previously described, to evaluate the expression and localization of AKNA (1:200, FNab00259, Fine-Test, China), STING (1:4000, 19851-1-AP, Proteintech, China), CD86 (1:500, 26903-1-AP, Proteintech, China), and CD206 (1:4000, 18704-1-AP, Proteintech, China). Immunostaining assessment was conducted independently by two pathologists. AKNA and STING expression levels were evaluated using a semi-quantitative scoring system. Tumor cells were classified into four intensity grades: 1 (negative), 2 (weak), 3 (moderate), and 4 (strong). The H-score was calculated as: H-score = (1 × P_1_) + (2 × P_2_) + (3 × P_3_) + (4 × P_4_), where P_1_–P_4_ represent the proportions of cells at each grade (summing to 1.0). This yields a continuous score ranging from 1.0 to 4.0. If estimates for any intensity category differed by > 10%, a third pathologist adjudicated. To ensure methodological harmonization across the two cohorts, a fixed H-score cutoff of ≥ 2.5, which corresponds to the natural midpoint of the semiquantitative scale and was established and externally validated in our previous study (23), was employed to define high expression. Quantification of CD86^+^/CD206^+^ macrophages was performed according to published protocols (24).

### 2.6 Western blotting

Western blotting was performed as previously described (24). Primary antibodies were as follows: AKNA (1:1000, 23785-1-AP, Proteintech, China), Ki-67 (1:500, sc-23900, Santa Cruz Biotechnology, USA), N-cadherin (1:10000, 82968-1-RR, Proteintech, China), E-cadherin (1:50000, 20874-1-AP, Proteintech, China), vimentin (1:20000, 80232-1-RR, Proteintech, China), cyclic GMP–AMP synthase (cGAS) (1:1000, PQA3430, Abmart, China), STING (1:50000, 19851-1-AP, Proteintech, China), phospho-STING (p-STING, 1:1000, PA5-105674, Thermo Fisher, USA), TANK-binding kinase 1 (TBK1) (1:1000, 28397-1-AP, Proteintech, China), phospho-TBK1 (p-TBK1, 1:2500, 61294, GenuIN, China), interferon regulatory factor 3 (IRF3) (1:2500, 61219, GenuIN, China), phospho-IRF3 (p-IRF3, 1:2000, 29528-1-AP, Proteintech, China), and GAPDH (1:50000, 60004-1-Ig, Proteintech, China).

### 2.7 Reverse transcription quantitative PCR (RT-qPCR)

Total RNA was extracted using the RNeasy Mini Kit (Qiagen, Hilden, Germany), and RNA concentration and purity were assessed spectrophotometrically. cDNA synthesis was performed using RT Master Mix (MedChemExpress, USA). RT-qPCR was performed on a Bio-Rad CFX96 system using SYBR Green PCR master mix, with GAPDH as the internal reference gene (24). Primer sequences were provided in **Supplementary Table 2**.

### 2.8 Cell culture

The human EC cell lines HEC-1A and Ishikawa, and the human monocytic leukemia cell line THP-1, were purchased from the Chinese Academy of Sciences Cell Bank. HEC-1A and Ishikawa cells were cultured in DMEM, high glucose (Gibco, USA) supplemented with 10% fetal bovine serum (FBS, Lonsera, China). THP-1 cells were maintained in RPMI 1640 (Gibco, USA) supplemented with 10% FBS and 50 μM β-mercaptoethanol. All cells were cultured at 37 °C in a humidified 5% CO_2_ atmosphere in a Thermo Scientific CO_2_ incubator, and the medium was changed every 2–3 days.

### 2.9 Lentiviral transfection

Lentiviral vectors for AKNA knockdown, AKNA overexpression (oeAKNA), and STING overexpression (oeSTING) were purchased from Genechem (China). HEC-1A and Ishikawa cells were plated in six-well plates and incubated for 24 h until they reached ~30% confluency. Transduction was performed at manufacturer-specified multiplicities of infection (20 for Ishikawa and 10 for HEC-1A) using the HitransG™ A enhancer. After replacement of the supernatant with fresh high-glucose DMEM supplemented with 10% FBS, stable pools were selected with 2 μg/mL puromycin for 14 days. Knockdown and overexpression efficiencies were validated by RT-qPCR and western blotting. The shRNA sequences were as follows: shAKNA-1, 5’-GCTCAGAAACAAGCAGAGTTT-3’; shAKNA-2, 5’-CCAATACACAGGCCACGAATA-3’.

### 2.10 Cell migration, invasion and proliferation assays

Cell migration was assessed using Transwell and scratch-wound assays. Transwell migration and invasion assays were performed using 8.0 μm pore-size chambers (Biosharp, China). For invasion assays, chamber membranes were pre-coated with Matrigel (BD Biosciences, USA) and incubated at 37°C for 1 h, whereas uncoated membranes were used for migration assays. HEC-1A and Ishikawa cells (6 × 10^4^) in serum-free medium were seeded into the upper chambers, and the lower chambers were filled with medium supplemented with 20% FBS. After incubation for 48 h, non-migratory cells on the upper surface were removed. Cells that had migrated to or invaded the lower surface were fixed with 4% paraformaldehyde and stained with 0.5% crystal violet.

For the scratch-wound assay, HEC-1A and Ishikawa cells were seeded in six-well plates and cultured in complete medium until they reached confluence. A linear scratch was created using a 200 μL pipette tip. After washing with PBS to remove cellular debris, cells were maintained in serum-free medium. Images of the scratch area were captured at 0, 24, and 48 h. Wound healing area was analyzed using ImageJ to quantify migration.

For the proliferation assay, HEC-1A and Ishikawa cells transfected with shNC, shAKNA, or oeAKNA were seeded in 96-well plates at 2 × 10^3^ cells per well and cultured for 24 h in a humidified incubator at 37 °C with 5% CO_2_. Cell Counting Kit-8 (CCK-8, Dojindo, Japan) was added at 0, 24, 48, and 72 h after seeding. After incubation for 1 h at 37 °C, absorbance was measured at 450 nm using a spectrophotometer.

### 2.11 Conditioned medium (CM) collection and macrophage polarization

For CM collection, EC cells from each group were cultured to approximately 80% confluency, gently washed with pre-warmed PBS to remove residual serum and metabolic byproducts, and replenished with an equal volume of fresh serum-free medium. Cells were then cultured for an additional 24 h at 37 °C in a humidified 5% CO_2_ atmosphere. CM was subsequently collected, centrifuged, and filtered through 0.22 μm membranes to completely remove tumor cells and debris (25, 26). For cyclic GMP–AMP (cGAMP)-treated groups, tumor cells were pulsed with 3 μM cGAMP (InvivoGen, France) for 4 h (27). To ensure that macrophages were not directly exposed to the STING agonist, the cGAMP-containing medium was subsequently removed, and the cells were washed with PBS. After replacement with fresh medium, cells were cultured for an additional 20 h before CM collection. The resulting cell-free CM was used to induce THP-1-derived macrophage polarization.

THP-1 monocytes were differentiated into M0 macrophages by treatment with 100 nM phorbol 12-myristate 13-acetate (PMA) (HY-18739, MedChemExpress, USA) for 24 h, followed by a 24 h resting period in fresh PMA-free complete medium (28). Differentiation was confirmed by adherence morphology under inverted microscopy (characteristic macrophage spreading and flattening). To exclude spontaneous polarization, baseline M1/M2 marker expression was assessed in unconditioned M0 controls using CD86-FITC (374204, BioLegend, USA) and CD206-PE (321106, BioLegend, USA); only batches with < 5% baseline CD86^+^ or CD206^+^ cells were utilized. Cells were subsequently washed three times with PBS to remove residual PMA. Macrophages were cultured in a 1:1 mixture of complete RPMI 1640 and CM for 48 h to maintain secretory profile and nutrient balance (18). Cells were stained with anti-human CD86-FITC (374204, BioLegend, USA) and CD206-PE (321106, BioLegend, USA) for 30 min at 4 °C in the dark. Unstained controls were included to establish baseline fluorescence. Flow cytometry was performed using a Beckman Coulter CytoFLEX S with CytExpert software. For analysis, cell debris was excluded based on FSC-A/SSC-A, followed by singlet gating using FSC-H/FSC-A. Positive/negative boundaries were determined using unstained controls. The detailed gating strategy is provided in **Supplementary Figure 1**.

### 2.12 Colony formation assay

HEC-1A and Ishikawa cells were seeded at 3,000 cells per well in six-well plates. All groups, including cGAMP-pretreated (3 μM) and untreated controls, were exposed to paclitaxel (HY-B0015, MedChemExpress, USA) or cisplatin (HY-17394, MedChemExpress, USA) for 14 days (29). Colonies were fixed (4% paraformaldehyde, 20 min), stained (0.1% crystal violet, 30 min), and counted when > 50 μm in diameter. The colony reduction ratio was calculated as: (colonies in the untreated group − colonies in the drug-treated group)/colonies in the untreated group.

### 2.13 ELISA

The concentrations of interferon-β (IFN-β), CXCL10, and CCL5 in CM from EC cells were measured using commercial ELISA kits (MULTI SCIENCES, Cat# EK1236, EK168, EK1139) according to the manufacturers’ protocols.

### 2.14 Chromatin immunoprecipitation (ChIP)

ChIP assays were performed using the Pierce Agarose ChIP Kit (Thermo Fisher Scientific) according to the manufacturer’s protocols. Briefly, cells were fixed in 1% formaldehyde for 10 min at room temperature, quenched with glycine buffer, and digested with micrococcal nuclease to generate DNA fragments. The resulting supernatant was divided into input and immunoprecipitation (IP) portions. For IP, samples were incubated overnight at 4 °C with 10 µg of anti-FLAG antibody (Cell Signaling Technology, #14793S) or IgG control, followed by 1 h incubation with Protein A/G Plus agarose. Immunoprecipitated complexes were eluted, and purified DNA fragments were normalized to % input for RT-qPCR analysis using primers targeting the predicted STING promoter binding sites (**Supplementary Table 2**).

### 2.15 Luciferase reporter assay

Wild-type (WT) and mutant (Mut) STING plasmid vectors were obtained from Genecreate Biotechnology. Luciferase reporter assays were performed following the manufacturer’s protocol using the Dual-Lumi™ Luciferase Reporter Gene Assay Kit (RG088S, Beyotime Biotechnology). Briefly, cells were seeded in 24 well plates at a density of 2 × 10^5^ cells per well and cultured for 24 h prior to transfection with the WT/Mut plasmids. At 12 h post-transfection, cells were replated into 96-well plates for a further 24 h. Firefly luciferase activity was measured using a multimode microplate reader after adding 100 μL detection reagent and incubating for 10 min. Subsequently, 100 μL Renilla luciferase detection reagent was added with the same incubation time to quantify Renilla luciferase signals. Firefly luciferase activity was normalized to Renilla luciferase activity to control for transfection efficiency.

### 2.16 Subcutaneous mouse model

After 1 week acclimation, 4-week-old female BALB/c nude mice (n = 30, 5 mice per group, Chengdu Yaokang Biotechnology) were used for two independent xenograft experiments as described below. First, to assess the tumor-suppressive role of AKNA and the rescue effect of STING, 1 × 10^6^ Ishikawa cells (shNC, shAKNA, or shAKNA + oeSTING transfectants) in 100 μL PBS were injected subcutaneously into the right thigh of mice (n = 15, 5 per group). From day 4 post-inoculation, tumor progression was monitored every 3 days by measuring tumor volume (V = 0.5 × L × W²) and body weight using a digital caliper. Second, to evaluate whether pharmacological STING activation can overcome chemoresistance in AKNA-deficient EC, 1 × 10^6^ shAKNA Ishikawa cells in 100 μL PBS were injected subcutaneously into the right thigh of mice (n = 15, 5 per group). Once tumors reached approximately 50 mm³, mice were randomly allocated to three groups: (1) Vehicle group, receiving PBS (intratumoral, every 3 days) and PBS (intraperitoneal, on Days 0, 3, and 7); (2) paclitaxel plus carboplatin (TP) group, receiving carboplatin (15 mg/kg) and paclitaxel (12 mg/kg) via intraperitoneal injection on Days 0, 3, and 7 (30); and (3) TP + cGAMP group, receiving intratumoral cGAMP (0.5 mg/kg, every 3 days) as adapted from established STING activation protocols (31), combined with the same TP regimen. Tumor volume and body weight were monitored every 3 days from day 4 post-inoculation. At study termination, all mice were anesthetized with sodium pentobarbital and euthanized by cervical dislocation, and tumors were excised and weighed. All animal procedures were approved by the Animal Ethics Committee and performed in strict accordance with institutional animal care and welfare guidelines.

### 2.17 Nomogram development and validation

A recurrence-predicting nomogram was developed using the training cohort (n = 395) and externally validated in an independent cohort (n = 285). Prognostic factors were first identified by univariate Cox regression (*P* < 0.05) and subsequently entered into multivariate Cox regression to determine independent predictors (*P* < 0.05). The nomogram was constructed using R software (v4.3.2; survival and rms packages) and incorporated the optimal 3-year RFS risk threshold, determined by maximizing Youden’s index on receiver operating characteristic (ROC) analysis of the training cohort (21). Decision curve analysis (DCA) was performed using the rms package. Net benefit was calculated as: Net benefit = (True positives/N) – (False positives/N) × (Pt/(1 – Pt)), where Pt represents the threshold probability.

### 2.18 Statistical analysis

Statistical analyses were performed using SPSS 26.0 (IBM), GraphPad Prism 8.0.2 (GraphPad), and R (v4.3.2). Continuous variables were expressed as mean ± standard deviation (SD) or median (interquartile range), as appropriate. Comparisons between groups were performed using Student’s t-test or one-way ANOVA for normally distributed data, and the Wilcoxon rank-sum test or Kruskal-Wallis test for non-normally distributed data. Categorical variables were reported as n (%) and compared using chi-square or Fisher’s exact test. Statistical significance was set at *P* < 0.05.

## 3 Results

### 3.1 Downregulation of AKNA correlates with poor prognosis in EC

Analysis of the TCGA pan-cancer data revealed significantly reduced AKNA expression in multiple solid tumors, notably in EC (**Figures 1A, B**). In EC, AKNA expression decreased progressively with higher histologic grade (**Figure 1C**) and advanced FIGO stage (**Figure 1D**), reaching minimal levels in the copy-number high subtype (**Figure 1E**). Protein profiling confirmed lower AKNA levels in EC than in normal endometrium (**Figure 1F**) and IHC data from our institutional cohort validated this finding (**Figure 1G**). This consistent downregulation at both mRNA and protein levels was corroborated in 20 matched tumor-normal pairs using IHC, RT-qPCR, and Western blotting (**Figures 1H–J**).

**Figure 1:**
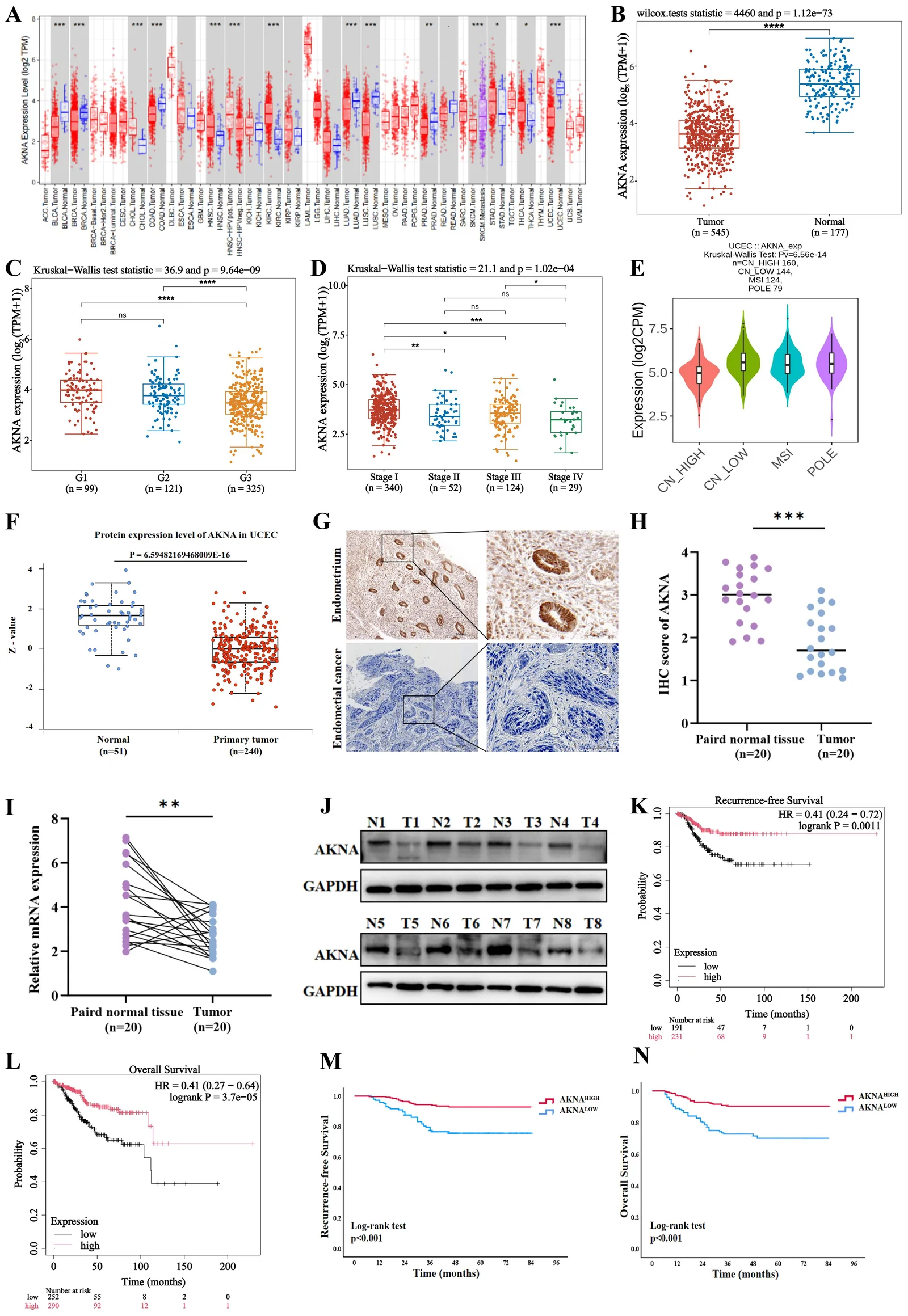
Expression profile and prognostic significance of AKNA in EC. **(A)** Pan-Cancer analysis of AKNA expression across the TCGA dataset. **(B)** Differential expression of AKNA between EC and normal endometrial tissues. **(C, D)** AKNA expression levels across distinct histological grades **(C)** and FIGO stages **(D)** of EC. **(E)** AKNA expression profiles across the TCGA-defined molecular subtypes of EC. **(F)** Protein level of AKNA in EC versus normal endometrial tissues. **(G)** Representative IHC staining of AKNA in normal endometrium and EC specimens. **(H)** Quantification of AKNA IHC scores in 20 paired tumor and adjacent non-tumor tissues from the institutional cohort. **(I)** RT-qPCR analysis of AKNA mRNA levels in 20 paired EC and adjacent non-tumor tissues. **(J)** Western blot analysis of AKNA protein expression in paired EC and adjacent normal tissues. **(K, L)** Kaplan-Meier survival curves for RFS **(K)** and OS **(L)** in the TCGA cohort, stratified by AKNA expression. **(M, N)** Kaplan-Meier curves for RFS **(M)** and OS **(N)** in the institutional cohort, stratified by AKNA expression. Data are presented as mean ± SD. *p < 0.05, **p < 0.01, ***p < 0.001, ****p < 0.0001; ns, not significant.

In the TCGA-UCEC cohort, low AKNA expression was significantly associated with advanced FIGO stage (*P* = 0.031), high tumor grade (*P* < 0.001), aggressive histological subtypes (*P* = 0.034), and TP53 mutation (*P* < 0.001) (**Supplementary Table 3**). These findings were validated in our institutional cohort, where reduced AKNA expression was correlated with older age (*P* = 0.033), advanced FIGO stage (*P* = 0.015), LVSI (*P* = 0.001), aberrant p53 expression (*P* = 0.023), and receipt of adjuvant therapy (*P* = 0.004) (**Supplementary Table 4**). Survival analysis revealed that high AKNA expression predicted favorable RFS and OS in both the TCGA-UCEC cohort (*P* = 0.0011 and *P* = 0.00037, respectively; **Figures 1K, L**) and our institutional cohort (both *P* < 0.001; **Figures 1M, N**).

### 3.2 AKNA suppresses malignant phenotypes in EC cells

Stable AKNA knockdown and overexpression cell lines were established via lentiviral transduction in both Ishikawa (**Supplementary Figures 2A, C**) and HEC-1A (**Supplementary Figures 2B, D**) cell lines. As shown by western blotting, silencing AKNA enhanced Ki-67 expression and promoted EMT in EC cells, as reflected by upregulated N-cadherin and vimentin, and downregulated E-cadherin. Conversely, AKNA overexpression suppressed these phenotypic changes (**Figures 2A, B**). CCK-8 assays demonstrated that downregulation of AKNA remarkably enhanced the proliferation in Ishikawa and HEC-1A cells, whereas AKNA upregulation attenuated cellular proliferative activity (**Figures 2C–F**). In scratch wound-healing assays, AKNA silencing accelerated gap closure, whereas its overexpression markedly delayed this process (**Figures 2G–J**). Similarly, transwell assays demonstrated that silencing AKNA promoted cell migration and invasion, whereas enforced AKNA expression inhibited these malignant phenotypes (**Figures 2K–N**). These results demonstrated that AKNA acts as a suppressor of proliferation, migration, and EMT in EC cells.

**Figure 2:**
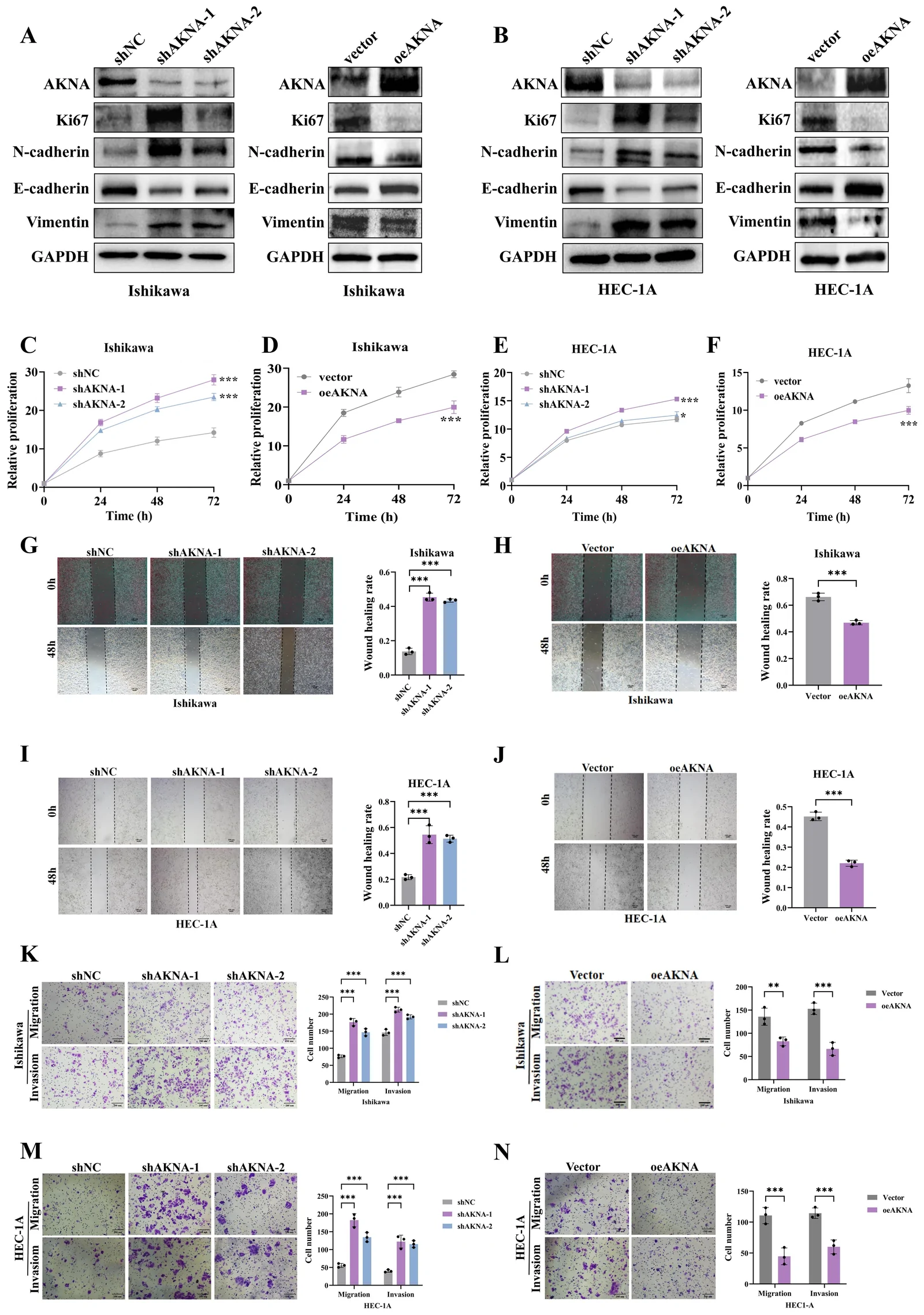
AKNA inhibits the proliferation, migration, and invasion of EC cells. **(A, B)** Western blot analysis of AKNA and EMT-related markers in Ishikawa **(A)** and HEC-1A **(B)** cells following AKNA knockdown or overexpression. **(C–F)** CCK-8 assays evaluating the proliferation of Ishikawa **(C, D)** and HEC-1A **(E, F)** cells after AKNA knockdown or overexpression. **(G–J)** Representative images and quantification of wound healing assays assessing the migratory capacity of Ishikawa **(G, H)** and HEC-1A **(I, J)** cells with AKNA knockdown or overexpression. **(K–N)** Transwell migration and invasion assays in Ishikawa **(K, L)** and HEC-1A **(M, N)** cells subjected to AKNA knockdown or overexpression. Data are presented as mean ± SD. *p < 0.05, **p < 0.01, ***p < 0.001, ****p < 0.0001; ns, not significant.

### 3.3 AKNA expression associates with macrophage polarization in the EC microenvironment

ESTIMATE algorithm analysis revealed that high-AKNA EC samples exhibited significantly increased stromal, immune, and ESTIMATE scores (all *P* < 0.01), together with reduced tumor purity (*P* < 0.0001), consistent with an immune-enriched tumor microenvironment (**Figures 3A, B**). AKNA expression was positively correlated with T cells (*P* = 3.08 × 10^-2^), myeloid dendritic cells (*P* = 1.71 × 10^-25^), monocytes (*P* = 2.92 × 10^-18^), and macrophages and monocytes (*P* = 2.92 × 10^-18^) as quantified by MCP-counter (**Figure 3C**). Given the well-established association between AKNA and T cells in previous studies, the present study focused on the potential correlation between AKNA and macrophages (32). Single-cell profiling revealed preferential AKNA enrichment in myeloid compartments (monocytes, macrophages, and dendritic cells; **Supplementary Figure 3A**), with concurrent enrichment in lymphoid subsets and fibroblasts (**Supplementary Figure 3B**). GSEA demonstrated significant activation of immune pathways in the high-AKNA group, including enhanced immune infiltration, innate immunity, and macrophage activation, differentiation, proliferation, and migration (all *P* < 0.001, **Figures 3D–I**). Extended analysis of the TCGA cohort confirmed positive correlations between AKNA and tumor-infiltrating lymphocyte markers, MHC class II molecules, and co-stimulatory molecules (**Supplementary Figures 4A–C**). Subsequent functional assays confirmed that conditioned medium from AKNA-knockdown EC cells promoted macrophage polarization toward an M2-like phenotype, as evidenced by a reduced proportion of CD86^+^ M1-like cells and an enhanced proportion of CD206^+^ M2-like cells (*P* < 0.001, **Figures 3J, L**). Conversely, medium from AKNA-overexpressing EC cells elevated CD86^+^ M1 and reduced CD206^+^ M2 macrophage proportions (*P* < 0.001, **Figures 3K, M**).

**Figure 3:**
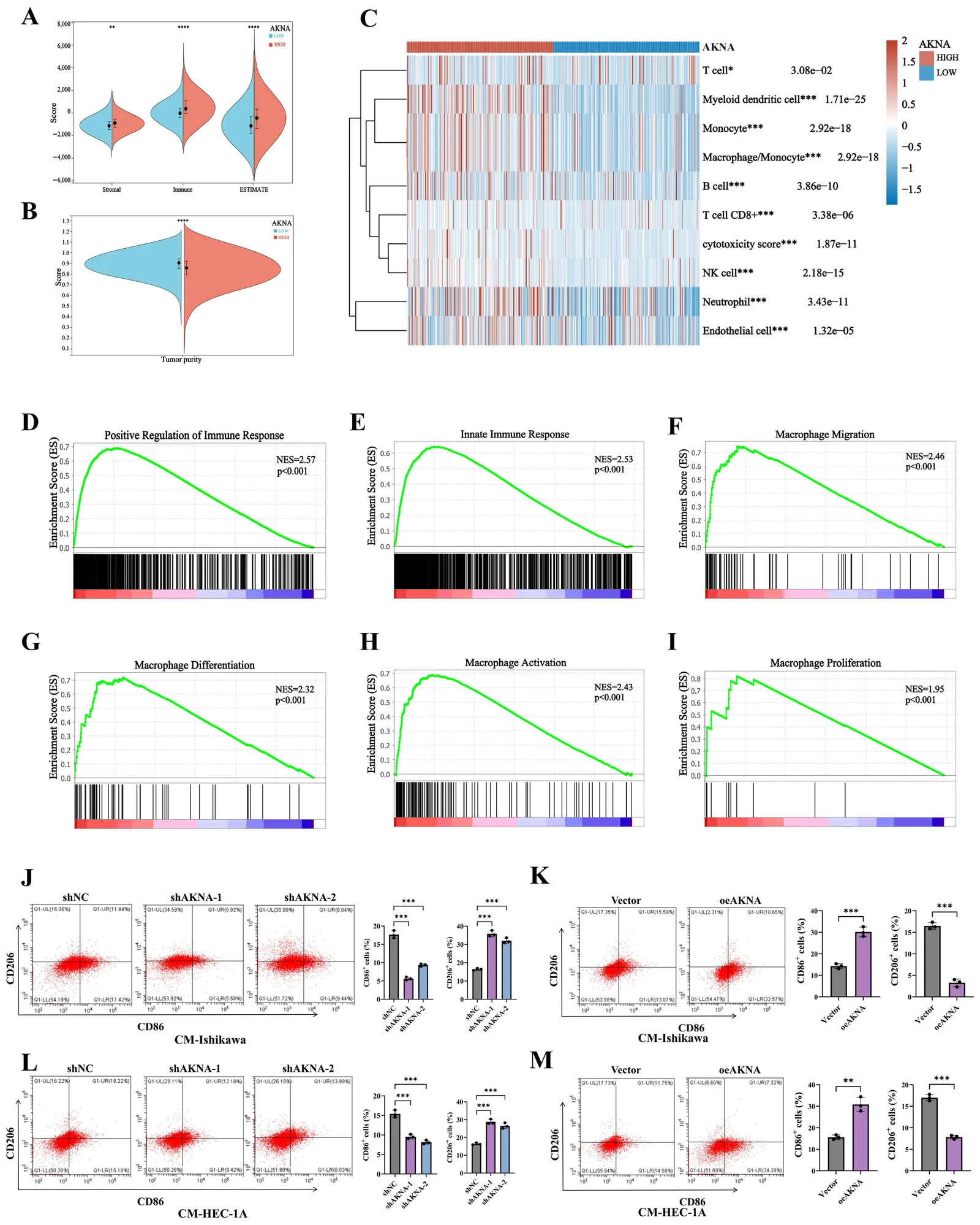
AKNA modulates the innate immune response and macrophage polarization in EC. **(A, B)** Correlation of AKNA expression with ESTIMATE, stromal, and immune scores **(A)**, and tumor purity **(B)** in the TCGA-UCEC cohort. **(C)** Correlation of AKNA expression with immune and stromal cell infiltration. **(D-I)** GSEA revealed enrichment of gene sets involved in positive regulation of immune responses, innate immune responses, and macrophage migration, differentiation, activation, and proliferation. **(J-M)** Flow cytometric analysis of CD86^+^ M1-like and CD206^+^ M2-like macrophages following treatment with conditioned medium from AKNA-knockdown and AKNA-overexpressing Ishikawa **(J, K)** and HEC-1A **(L, M)** cells. Data are shown as mean ± SD. *p < 0.05, **p < 0.01, ***p < 0.001, ****p < 0.0001; ns, not significant.

### 3.4 AKNA mediates macrophage polarization via cGAS-STING pathway in EC

GeneMANIA predicted AKNA interactions with immune effectors including ITGAL, CD38, and LAX1 (**Supplementary Figure 5A**), which is consistent with positive correlations of AKNA with immune-related genes (ITGAL, GPR18, P2RY13) in TCGA-UCEC (**Supplementary Figure 5B**). GO/KEGG analyses confirmed enrichment of AKNA-coexpressed genes in immune-inflammatory processes, particularly innate immunity, cytosolic DNA sensing, and JAK-STAT signaling (**Figures 4A, B**). Given the central role of the cGAS-STING pathway in cytosolic DNA sensing and innate immunity (33), we investigated AKNA’s impact on this pathway. AKNA differentially regulated STING signaling. AKNA knockdown reduced phosphorylation of TBK1, IRF3, and STING, decreased STING protein abundance without affecting total TBK1 and IRF3 levels, whereas its overexpression enhanced phosphorylation of these proteins and selectively increased STING abundance (**Figures 4C, D**). Mechanistically, STING overexpression or cGAMP treatment restored TBK1 and IRF3 phosphorylation in AKNA-knockdown cells (**Figures 4E, F**). Critically, CM from AKNA-knockdown EC cells polarized macrophages toward immunosuppressive CD206^+^ M2 phenotypes. This shift was reversed by either STING overexpression or cGAMP stimulation (**Figures 4G–J**).

**Figure 4:**
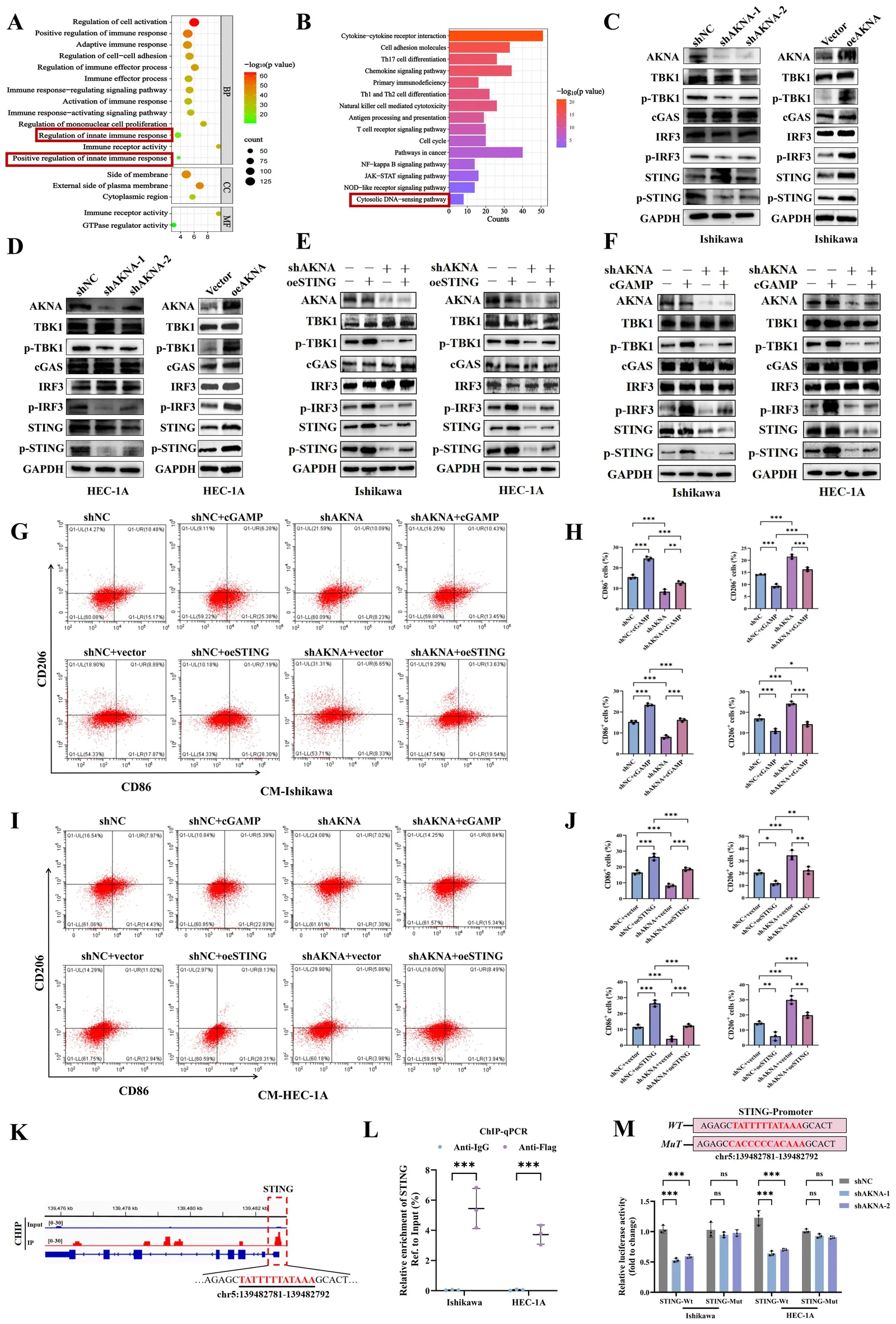
The functional role of AKNA in regulating STING signaling in EC cells. **(A)** GO term enrichment analysis of AKNA-coexpressed genes. **(B)** KEGG pathway enrichment analysis of AKNA-coexpressed genes. **(C, D)** Western blot analysis of AKNA, cGAS, and total and phosphorylated STING, TBK1, and IRF3 in Ishikawa **(C)** and HEC-1A **(D)** cells following AKNA knockdown and AKNA overexpression. **(E)** Western blot analysis of cGAS, and total and phosphorylated STING, TBK1, and IRF3 in AKNA-knockdown EC cells ± STING overexpression. **(F)** Western blot analysis of cGAS, and total and phosphorylated STING, TBK1, and IRF3 in AKNA-knockdown EC cells ± cGAMP treatment. **(G–J)** Flow cytometry analysis of CD86^+^ (M1-like) and CD206^+^ (M2-like) subsets in M0 macrophages cultured with conditioned media from Ishikawa **(G, H)** or HEC-1A **(I, J)** cells under AKNA-knockdown/overexpression ± cGAMP or ± oeSTING. **(K)** AKNA ChIP-seq binding peaks at the STING promoter. **(L)** ChIP-qPCR analysis showing AKNA binding to the STING promoter in EC cells. **(M)** Dual-luciferase reporter assays in Ishikawa and HEC-1A cells. Note: Data are presented as mean ± SD. *p < 0.05, **p < 0.01, ***p < 0.001, ****p < 0.0001; ns, not significant.

To elucidate the secretory mediators connecting AKNA-mediated STING activation to macrophage polarization, ELISA was performed on CM derived from EC cells. AKNA knockdown markedly reduced the secretion of IFN-β and CXCL10, whereas AKNA overexpression significantly elevated their levels in both Ishikawa and HEC-1A cells (**Supplementary Figures 6A, B**). CCL5 secretion remained comparable across all groups, with no statistically significant differences (**Supplementary Figure 6C**). However, CXCL9 and CXCL11 were consistently below the lower limit of quantification. Collectively, these data demonstrate that the AKNA–STING axis selectively governs the IFN-β and CXCL10 secretory phenotype in EC cells to regulate macrophage polarization.

Given prior reports of AKNA binding to the promoters of target genes (32), we performed AKNA ChIP-seq in Ishikawa cells. Strikingly, distinct binding peaks were observed at the STING transcription start site, which co-localized with A/T-rich regulatory clusters (**Figure 4K**). ChIP-qPCR validation confirmed significant AKNA enrichment at the STING promoter in the anti-Flag IP group compared with the control IgG group (**Figure 4L**). Functional analysis via luciferase reporter assays demonstrated that mutagenesis of these A/T-rich elements within the STING promoter abolished AKNA-mediated transcriptional activation (**Figure 4M**).

In subcutaneous Ishikawa xenografts, tumor volume and tumor growth rate were significantly increased in the AKNA-knockdown group compared with the control group, and this phenotype was partially rescued by STING overexpression (**Figures 5A–C**). IHC revealed that tumors with AKNA knockdown exhibited an immunosuppressive microenvironment characterized by reduced STING expression, decreased CD86^+^ cell infiltration, and increased CD206^+^ accumulation, whereas STING overexpression partially rescued this phenotype (all *P* < 0.05, **Figures 5D–H**). Intratumoral AKNA expression was positively correlated with both STING levels (*r* = 0.5611, *P* = 0.0013) and CD86^+^ cell density (*r* = 0.6131, *P* = 0.0006), and inversely correlated with CD206^+^ density (*r* = -0.7134, *P* < 0.001, **Figures 5I–K**). Analysis of 395 clinical EC specimens revealed that diminished AKNA expression was significantly associated with reduced STING protein levels, decreased CD86^+^ cell density, and increased CD206^+^ cell accumulation (all *P* < 0.0001, **Figures 6A–D**). Further correlation analysis confirmed positive associations between AKNA expression and both STING expression (*r* = 0.44) and CD86^+^ cell density (*r* = 0.55), together with an inverse correlation with CD206^+^ cell density (*r* = -0.56, all *P* < 0.0001, **Figures 6E–G**).

**Figure 5:**
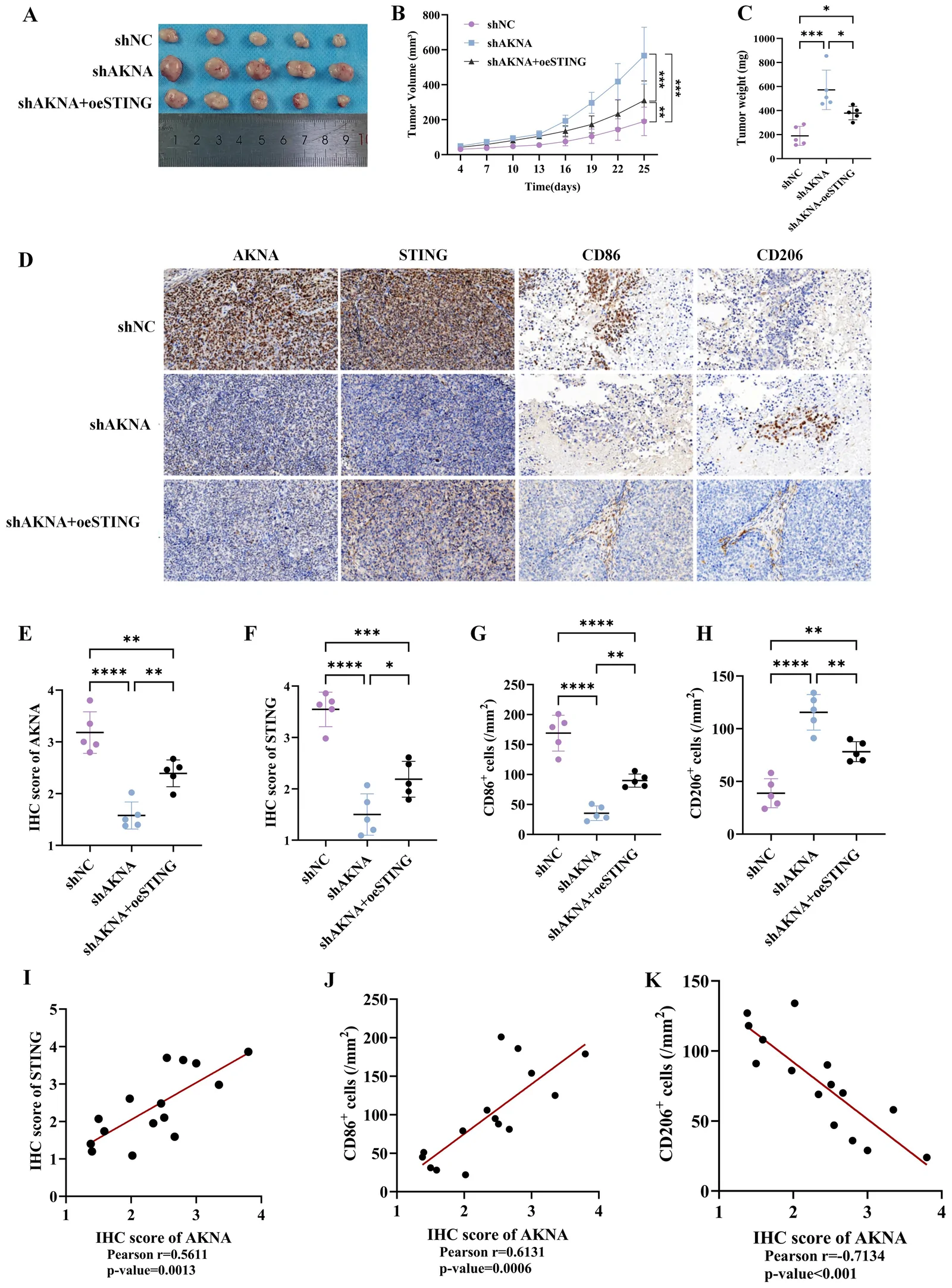
AKNA knockdown promotes tumor growth and modulates macrophage polarization by regulating the STING signaling pathway in EC *in vivo*. **(A)** Xenograft tumors from shNC, shAKNA and shAKNA + oeSTING groups. **(B)** Tumor growth curves. **(C)** Tumor weights at the experimental endpoint. **(D)** Representative IHC staining for AKNA, STING, CD86, and CD206. **(E–H)** IHC-based quantification of AKNA **(E)**, STING **(F)**, CD86^+^ M1-like macrophage infiltration **(G)**, and CD206^+^ M2-like macrophage infiltration **(H)** in tumor tissues. **(I–K)** Correlation analysis between AKNA expression and STING IHC scores **(I)**, CD86^+^ cell density **(J)**, and CD206^+^ cell density **(K)** across all tumor samples. Data are presented as mean ± standard deviation (SD). *p < 0.05, **p < 0.01, ***p < 0.001, ****p < 0.0001; ns, not significant.

**Figure 6:**
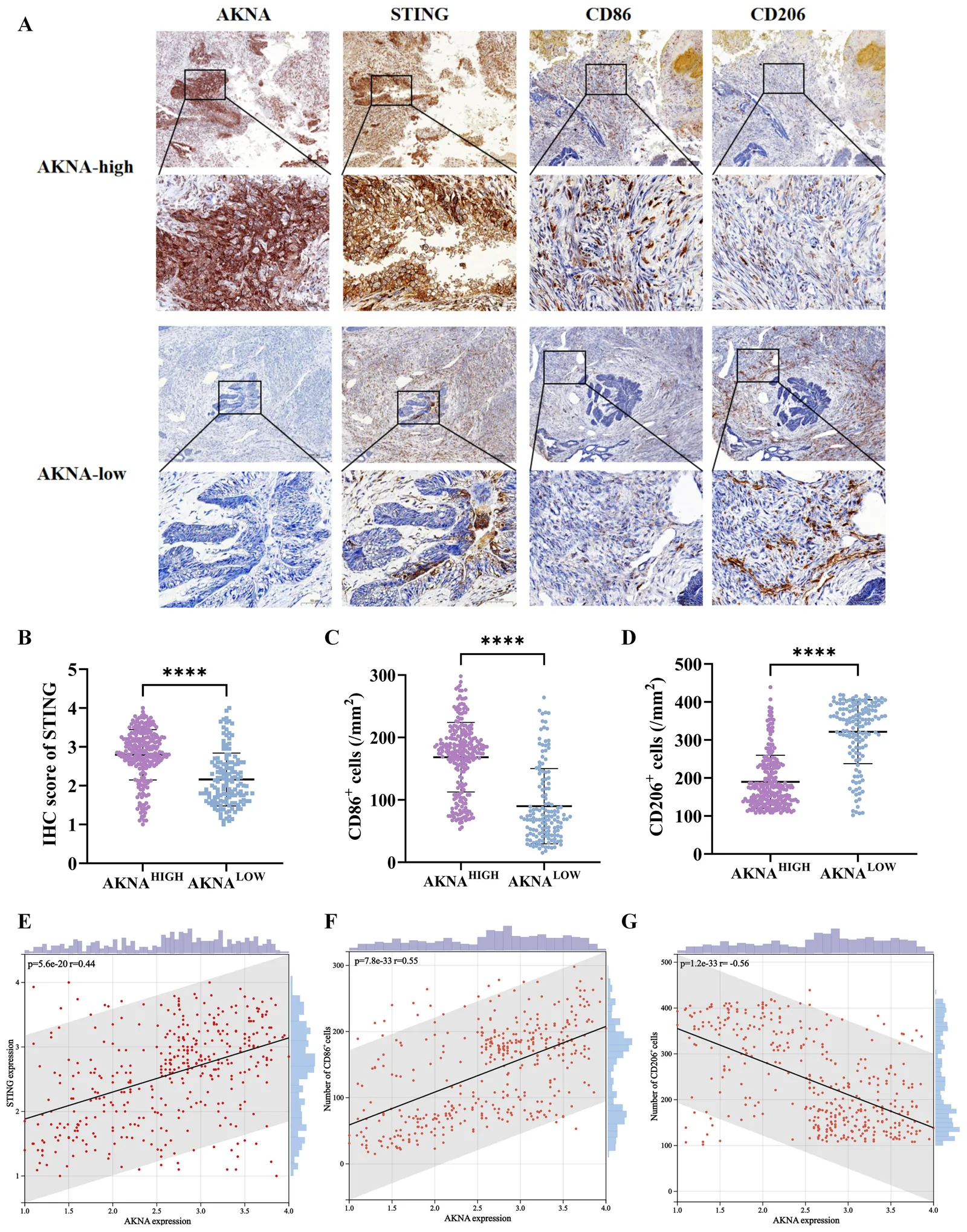
Association of AKNA expression with STING levels and macrophage polarization in clinical EC specimens. **(A)** Representative IHC images of STING, CD86, and CD206 in EC tissues stratified by AKNA-high and AKNA-low expression. **(B–D)** Quantitative analysis of STING expression **(B)**, CD86^+^ cell density **(C)**, and CD206^+^ cell density **(D)** in AKNA-high versus AKNA-low groups. **(E–G)** Correlation analysis between AKNA expression and STING levels **(E)**, CD86^+^ cell density **(F)**, and CD206^+^ cell density **(G)**. Data are presented as mean ± SD. *P < 0.05, **P < 0.01, ***P < 0.001, ****P < 0.0001; ns, not significant.

### 3.5 Bioinformatic prediction of immunotherapy response and validation of chemosensitivity

Using the GDSC database, we demonstrated that high AKNA expression was significantly associated with increased sensitivity to paclitaxel (*P* < 0.05) and docetaxel (*P* < 0.05) (**Figures 7A, B**). In addition, TIDE algorithm analysis of TCGA-UCEC patients revealed that the proportion of predicted immunotherapy responders was 11.3% higher in high-AKNA EC than in low-AKNA EC (42.9% versus 31.6%, *P* = 0.065) (**Figures 7C, D**). Based on this bioinformatic prediction, high-AKNA EC may be more responsive to immunotherapy.

**Figure 7:**
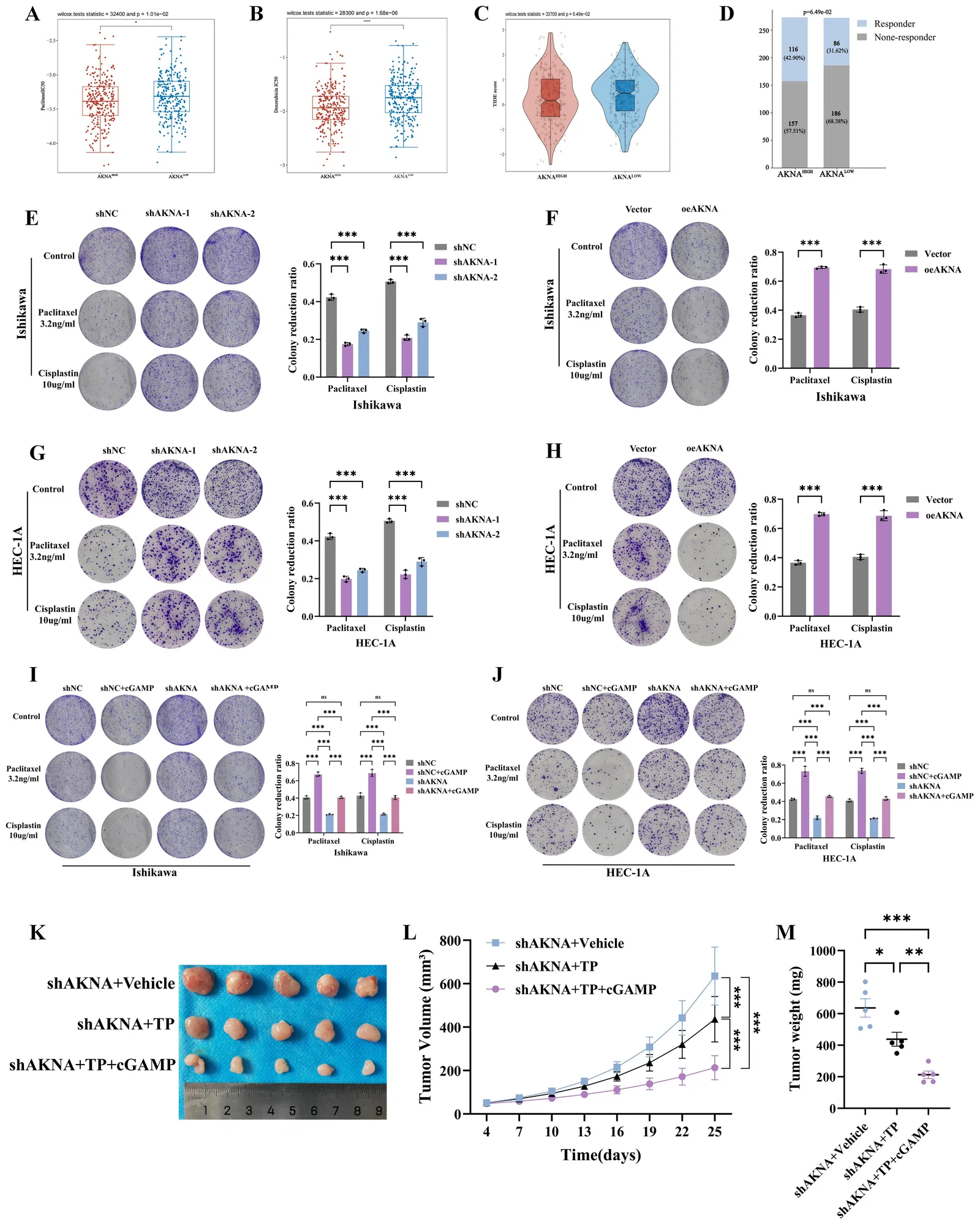
AKNA regulates chemotherapy sensitivity and tumor growth through the STING signaling pathway in EC. **(A, B)** Drug sensitivity analysis for paclitaxel **(A)** and docetaxel **(B)** in high-versus-low AKNA subgroups from the GDSC database. **(C, D)** TIDE-based bioinformatic analysis, showing TIDE scores **(C)** and responder distribution **(D)** in high- AKNA versus low-AKNA subgroups. **(E–H)** Colony formation assays in AKNA knockdown or AKNA overexpressing Ishikawa **(E, F)** and HEC-1A **(G, H)** cells following chemotherapy treatment. **(I, J)** Effect of the STING agonist cGAMP on colony formation in the shNC- or shAKNA-transduced Ishikawa **(I)** and HEC-1A **(J)** cells after chemotherapy treatment. **(K–M)** *In vivo* validation of therapeutic efficacy: tumor images **(K)**, tumor growth curves **(L)**, and final tumor weights **(M)** in shAKNA+Vehicle, shAKNA + TP, and shAKNA + TP + cGAMP groups. Note: Data are shown as mean ± SD. *p < 0.05, **p < 0.01, ***p < 0.001, ****p < 0.0001; ns, not significant.

Colony reduction ratio analysis demonstrated that the colony reduction ratio was significantly lower in shAKNA than in shNC EC cells following paclitaxel (3.2 ng/mL) or cisplatin (10 μg/mL) treatment, indicating that AKNA depletion reduced chemosensitivity at these specific concentrations (**Figures 7E, G**). Conversely, AKNA overexpression significantly increased the colony reduction ratio under these treatments, demonstrating enhanced chemosensitivity at these specific doses (**Figures 7F, H**). To determine whether AKNA regulates chemosensitivity through the STING pathway, we assessed the effect of cGAMP. cGAMP treatment significantly elevated the colony reduction ratio in shNC cells, indicating further enhanced chemosensitivity. Critically, cGAMP partially restored chemosensitivity at these concentrations in shAKNA cells, as evidenced by a significantly increased colony reduction ratio compared with untreated shAKNA cells (**Figures 7I, J**). These results indicated that AKNA modulated chemosensitivity to paclitaxel and cisplatin at the tested concentrations via the STING pathway.

To evaluate the therapeutic interaction between STING activation and chemotherapy in AKNA-deficient tumors, *in vivo* analysis was performed using shAKNA xenograft models. Compared with the vehicle group, TP monotherapy partially attenuated tumor growth, as reflected by significantly delayed tumor progression and reduced terminal tumor volume and weight (*P* < 0.05). Critically, combined treatment with cGAMP and TP resulted in markedly stronger tumor suppression than TP monotherapy alone (*P* < 0.05, **Figures 7K–M**), indicating that pharmacological activation of STING synergizes with TP chemotherapy to achieve superior tumor suppression.

### 3.6 Establishing a recurrence prediction model incorporating AKNA and clinicopathological factors

To establish and validate a prognostic model incorporating AKNA expression, the FAHCQMU cohort (*n* = 395) served as the training set, and the WCHCQMU cohort (*n* = 285) served as the validation set. Both cohorts demonstrated comparable baseline characteristics across all clinicopathological parameters (*P* > 0.05, **Supplementary Table 5**). Multivariable analysis was performed in the training cohort, incorporating factors identified as significant in univariate screening (**Supplementary Figure 7A**). This analysis confirmed AKNA as an independent predictor of EC recurrence. Critically, elevated AKNA expression was significantly associated with prolonged RFS and exerted a pronounced protective effect (hazard ratio = 0.363, 95% confidence interval: 0.207-0.643, *P* < 0.001). Additional established prognosticators, including FIGO stage, histological subtype, LVSI, p53 status, and depth of myometrial invasion, also remained statistically significant (all *P* < 0.05, **Supplementary Figure 7B**).

The combined prognostic model integrating AKNA expression and clinicopathological features showed superior predictive performance compared with single-variable models. The integrated model achieved an area under the curve (AUC) of 0.880 in the training cohort, outperforming AKNA alone (AUC = 0.659) and clinicopathological factors alone (AUC = 0.779) (**Supplementary Figure 8A**). This superior performance was maintained in the validation cohort, with AUCs of 0.872, 0.655, and 0.748 for the integrated model, AKNA alone, and clinicopathological factors alone, respectively (**Supplementary Figure 8B**). Within the resulting six-variable nomogram, AKNA contributed substantial prognostic value (**Figure 8A**). The nomogram showed good calibration at 1-, 3-, and 5-year intervals in both internal and external validation (**Figures 8B–G**). To evaluate the clinical utility of the predictive models, DCA was performed. In DCA, the nomogram showed negligible net benefit for 1-year RFS, but positive and superior net benefit across a broad range of threshold probabilities at 3- and 5-year RFS compared with clinicopathological parameters alone and AKNA alone. Similar trends were observed in both cohorts (**Supplementary Figure 9**). Using the optimal risk threshold (0.810) established by maximizing Youden’s index for 3-year recurrence prediction in ROC analysis (**Supplementary Figure 10A**), patients were stratified into low-risk (> 0.810) and high-risk (≤ 0.810) groups. Kaplan-Meier analysis verified significantly prolonged RFS and OS in low-risk patients in both the training (**Supplementary Figures 10B, C**) and validation cohorts (**Supplementary Figures 10D, E**).

**Figure 8:**
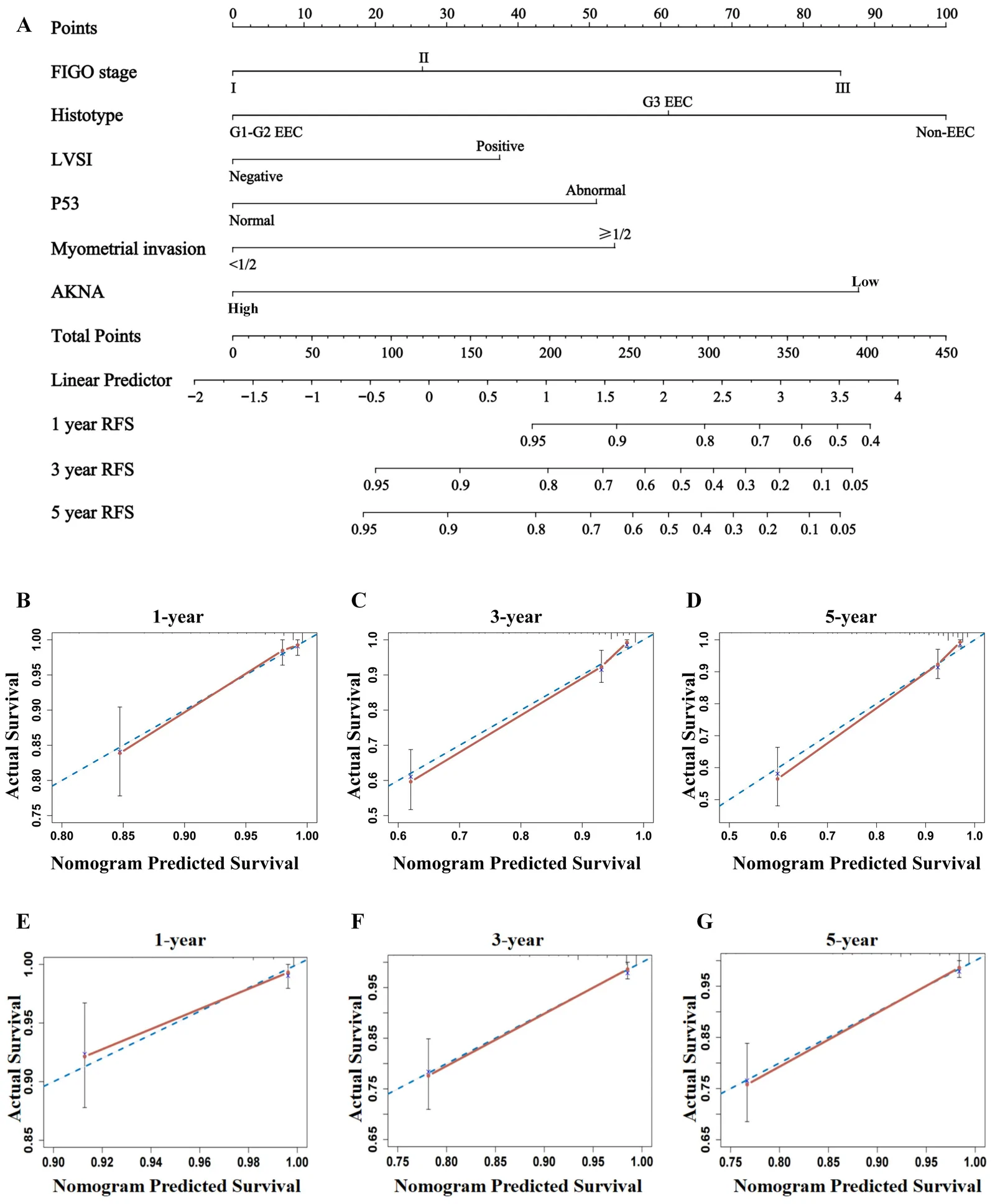
Construction and validation of the AKNA-based prognostic nomogram. **(A)** A prognostic nomogram integrating FIGO stage, histological subtype, LVSI, p53 status, depth of myometrial invasion, and AKNA expression for RFS prediction. **(B–G)** Calibration plots comparing predicted versus observed RFS probabilities at 1, 3, and 5 years in the training **(B–D)** and validation **(E–G)** cohorts. The blue dashed line represents the ideal performance of a perfectly calibrated model.

## 4 Discussion

The application of immune checkpoint inhibitors (ICIs) in EC is hindered by tumor heterogeneity, and only a subset of patients achieves favorable responses. Accordingly, the identification of reliable immunotherapeutic targets remains an urgent priority (34). This study demonstrates that AKNA expression is significantly reduced in EC tissues compared with matched normal endometrium. Critically, patients with low AKNA expression exhibit significantly poorer prognosis. These findings collectively suggest that AKNA expression may serve as a potential independent prognostic biomarker for EC. Functional validation experiments revealed that AKNA knockdown significantly enhances the proliferation, migration, and invasive capabilities of EC cells. This functional evidence strongly supports a tumor-suppressive role of AKNA in EC. Notably, the tumor-suppressive function of AKNA observed in EC aligns with its documented anti-tumorigenic roles in other carcinomas, including gastric cancer and cervical cancer (8, 11). This cross-tumor consistency further supports AKNA as a pan-cancer tumor suppressor.

AKNA has been implicated in immune-related biological processes in head and neck squamous cell carcinoma and lung adenocarcinoma (35, 36). Prior research has established AKNA as a pivotal master regulator in T-cell activation (6, 37). The present study investigated the association between AKNA expression and the immune microenvironment in EC. Our results revealed that AKNA expression is positively correlated with macrophage infiltration, stromal scores, and immune scores in EC. These indices represent key indicators for quantifying non-tumor stromal activity and have significant prognostic implications. GSEA of TCGA-UCEC data indicated enrichment of macrophage-related functional pathways in the high-AKNA group. Consequently, we performed functional validation and demonstrated that AKNA knockdown in EC cells promoted M2 macrophage polarization. These findings suggest that AKNA deficiency may promote EC progression by modulating macrophage polarization.

In the present study, we further demonstrated that AKNA suppresses M2 macrophage polarization by activating the cGAS-STING pathway, thereby promoting innate anti-tumor immunity. Accumulating evidence indicates that the cGAS-STING pathway modulates the activation, infiltration, and phenotypic switching of immune cells, including macrophages and T cells (38). The cGAS-STING pathway is a key component in triggering anti-tumor immune responses (39, 40). Previous studies have shown that IL-6 stimulation activates the cGAS-STING pathway in EC, contributing to tumor immune regulation (40). However, most previous studies have focused on cGAS-STING activation via conformational changes in cGAS (41, 42). In contrast, our study verified that AKNA could directly activate STING at the transcriptional level. Studies have reported that transcription factors such as HDAC3, HIF-1, NF-κB and STAT1 can also directly bind to the STING promoter and upregulate its expression, which is consistent with our findings (43–46). Collectively, these findings indicate that targeting AKNA-mediated regulation of STING may represent a promising therapeutic strategy for EC. While canonical cGAS-STING signaling requires cytosolic dsDNA as the initiating trigger, cancer cells inherently harbor genomic instability and micronuclei that generate endogenous cGAS ligands (47, 48). Under physiological STING levels, these subthreshold DNA signals fail to trigger robust downstream activation; however, by transcriptionally upregulating STING, AKNA expands the cellular pool of STING protein, effectively lowering the activation threshold and enabling efficient propagation of basal DNA damage signals into TBK1/IRF3 phosphorylation. This “transcriptional priming” model is consistent with our observation that AKNA overexpression alone enhances p-STING, p-TBK1, and p-IRF3 without exogenous DNA stimulation, and aligns with established evidence that chromosomal instability and endogenous DNA damage chronically engage the cGAS-STING axis in cancer cells (49).

Our CM transfer design holds macrophage-intrinsic AKNA constant, thereby isolating the tumor cell-autonomous effect; however, dissecting the relative contribution of myeloid AKNA represents an important future direction. Furthermore, mechanistic dissection of the secretome revealed that the AKNA–STING axis selectively promotes IFN-β and CXCL10 secretion in EC cells. By contrast, CCL5 secretion was unaltered, and CXCL9 and CXCL11 levels were below the lower limit of quantification. This selective induction of soluble mediators aligns with the canonical STING-TBK1-IRF3 signaling cascade, in which activated IRF3 preferentially drives transcription of type I interferons and interferon-stimulated genes (50). IFN-β and CXCL10 are well-characterized mediators facilitating M1 macrophage recruitment and activation (51, 52). The diminished secretion of these two factors following AKNA knockdown provides a mechanistic explanation for the M2-skewed macrophage polarization observed in our co-culture system. The absence of robust regulation of CXCL9, CXCL11 and CCL5 further highlights that AKNA-dependent STING activation directs macrophage polarization with marked specificity. While the present findings establish a tumor cell-autonomous paracrine axis, it is worth noting that AKNA is also enriched in myeloid populations. Our CM transfer design holds macrophage-intrinsic AKNA constant across groups, which isolates the tumor-derived signal as the operative variable. Nevertheless, macrophage-intrinsic AKNA may modulate the responsiveness to these paracrine cues, and dissecting its relative contribution represents an important future direction.

Although ICIs and TP combination chemotherapy represent important therapeutic strategies in EC, their clinical efficacy is hampered by suboptimal objective response rates and acquired drug resistance (53, 54). Based on predictive algorithmic models, our study suggests that high AKNA expression might correlate with superior therapeutic responses to ICIs in EC patients. Furthermore, we found that AKNA deficiency attenuates chemosensitivity to paclitaxel and cisplatin at the specific concentrations by attenuating TP-induced growth inhibition, a phenotype that can be partially rescued by the STING agonist cGAMP. In the shAKNA xenograft model, the combination of cGAMP and TP yielded significantly stronger tumor suppression than TP monotherapy in this experimental setting, indicating that pharmacological STING activation has the potential to mitigate AKNA-deficiency-driven chemoresistance *in vivo.* It should be noted that the *in vitro* chemosensitivity data were derived from single-dose colony formation assays. Future studies employing multi-concentration dose-response analyses are warranted to fully define the concentration-dependent effects of AKNA modulation on drug sensitivity. Consistent with these observations, prior studies have suggested that integrating STING agonists with conventional chemotherapy can sensitize tumors to immune-mediated clearance (55). Notably, by incorporating AKNA expression with established clinicopathological parameters, we developed a novel prognostic nomogram that enables individualized recurrence risk assessment and robust risk stratification. DCA curves further revealed that the integrated nomogram offered minimal net benefit for 1-year RFS, likely attributable to the inherently low early-event rate in EC that limits the clinical utility of any predictive model at this timepoint. In contrast, the nomogram demonstrated substantial net benefit at 3- and 5-year timepoints across a broad range of threshold probabilities. Notably, these patterns were consistent in both the training and validation cohorts, supporting the robustness and generalizability of the model for intermediate- to long-term risk stratification. Of note, this nomogram was developed and validated in patients with FIGO Stage I–III disease who underwent primary curative surgery, reflecting the clinical population most relevant to adjuvant therapy decision-making. While TCGA-UCEC data confirm that AKNA expression is lowest in Stage IV, supporting biological coherence across the full disease spectrum, the model’s direct applicability to advanced metastatic disease remains to be established. Future studies incorporating Stage IV patients with biopsy-accessible tumor tissue are warranted. Collectively, these findings underscore the dual translational significance of AKNA as both a sensitizing therapeutic target and a reliable prognostic biomarker in EC.

In summary, our study reveals that AKNA functions as a tumor suppressor with potent innate immunomodulatory properties in EC. Nevertheless, several limitations should be acknowledged. First, while IFN-β and CXCL10 were identified as key STING-dependent messengers in the AKNA-regulated secretome, the complete chemokine network and additional paracrine mediators remain to be fully characterized in future studies. Second, although promising, our AKNA-based recurrence prediction model warrants prospective validation in large-scale, multicenter clinical cohorts to confirm its clinical utility. Third, while the nude mouse model effectively validates the innate AKNA-STING-macrophage axis, its lack of mature T cells limits the direct evaluation of adaptive immunity and immune checkpoint inhibitors. Future studies using immunocompetent syngeneic models are warranted to fully confirm AKNA’s immunotherapeutic potential.

## 5 Conclusion

This study identifies AKNA as an independent prognostic biomarker associated with malignant progression and unfavorable clinical outcomes in EC. Mechanistically, AKNA directly binds to A/T-rich elements in the STING promoter to activate TBK1/IRF3 signaling, with restoration of this axis by cGAMP reversing malignant phenotypes both *in vitro* and *in vivo*. Clinically, AKNA serves as an independent prognostic biomarker, and its integration into a nomogram enables accurate recurrence prediction, offering a promising therapeutic target for modulating innate immunity and enhancing chemotherapy sensitivity in EC.

## Data Availability

The datasets presented in this study can be found in online repositories. The names of the repository/repositories and accession number(s) can be found below: https://www.ncbi.nlm.nih.gov/, TCGA-UCEC/GSE154763/GSE139555.
