# Supplementary material for: AKNA serves as a prognostic biomarker and restrains endometrial cancer progression via STING activation and macrophage reprogramming
Source: Front Immunol. 2026 Oct 5;17:1905624. doi: 10.3389/fimmu.2026.1905624 (PMC13677899; doi:10.3389/fimmu.2026.1905624)
Supplement: Supplementary file 1 [file DataSheet1.pdf]

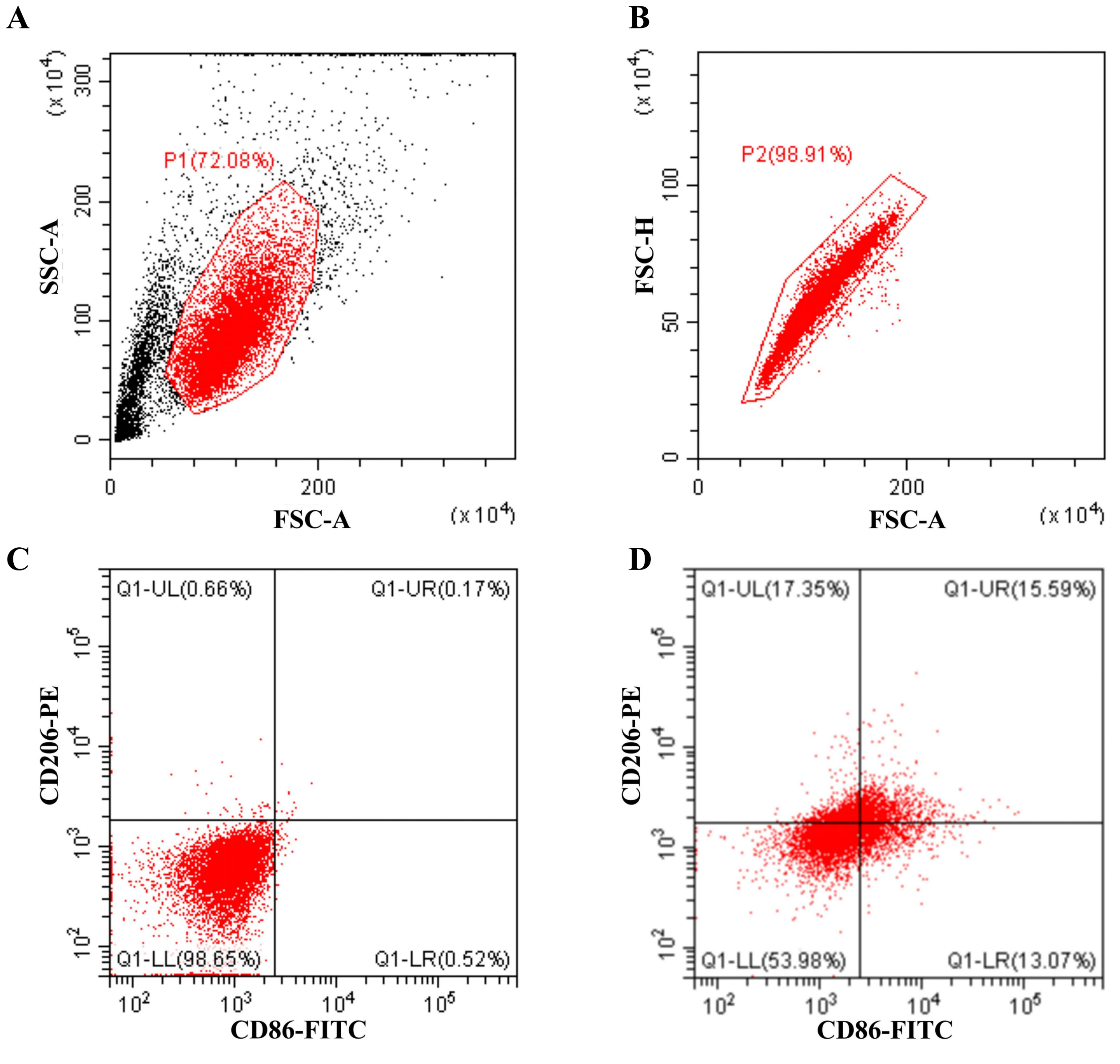

Supplementary Figure 1

Gating strategy for macrophage polarization by flow cytometry.

(A) Main cell population gated on FSC-A/SSC-A (P1). (B) Single cells gated on FSC-A/FSC-H within P1 (P2). (C) Unstained control establishing quadrant boundaries for CD86-FITC and CD206-PE. (D) Experimental sample quantifying CD86^{+} (M1-like), CD206^{+} (M2-like)macrophages.

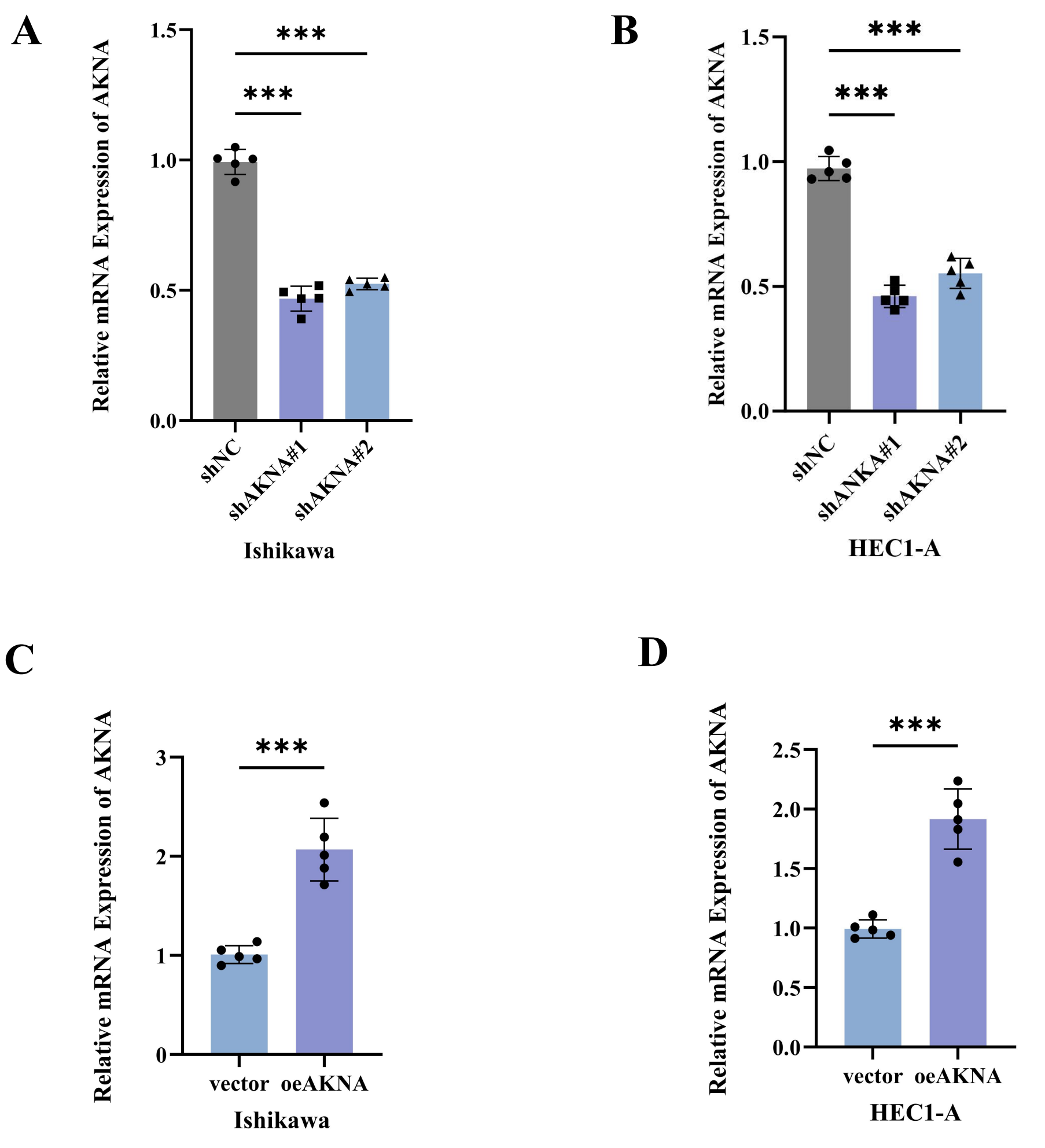

Supplementary Figure 2

Validation of AKNA and STING expression following lentiviral transduction in Ishikawa and HEC-1A cells. (A-D) RT-qPCR analysis of AKNA mRNA levels in Ishikawa and HEC-1A cells following knockdown (A, B) or overexpression (C, D). Data are presented as mean ± SD. *P< 0.05, **P< 0.01, ***P < 0.001; ns, not significant.

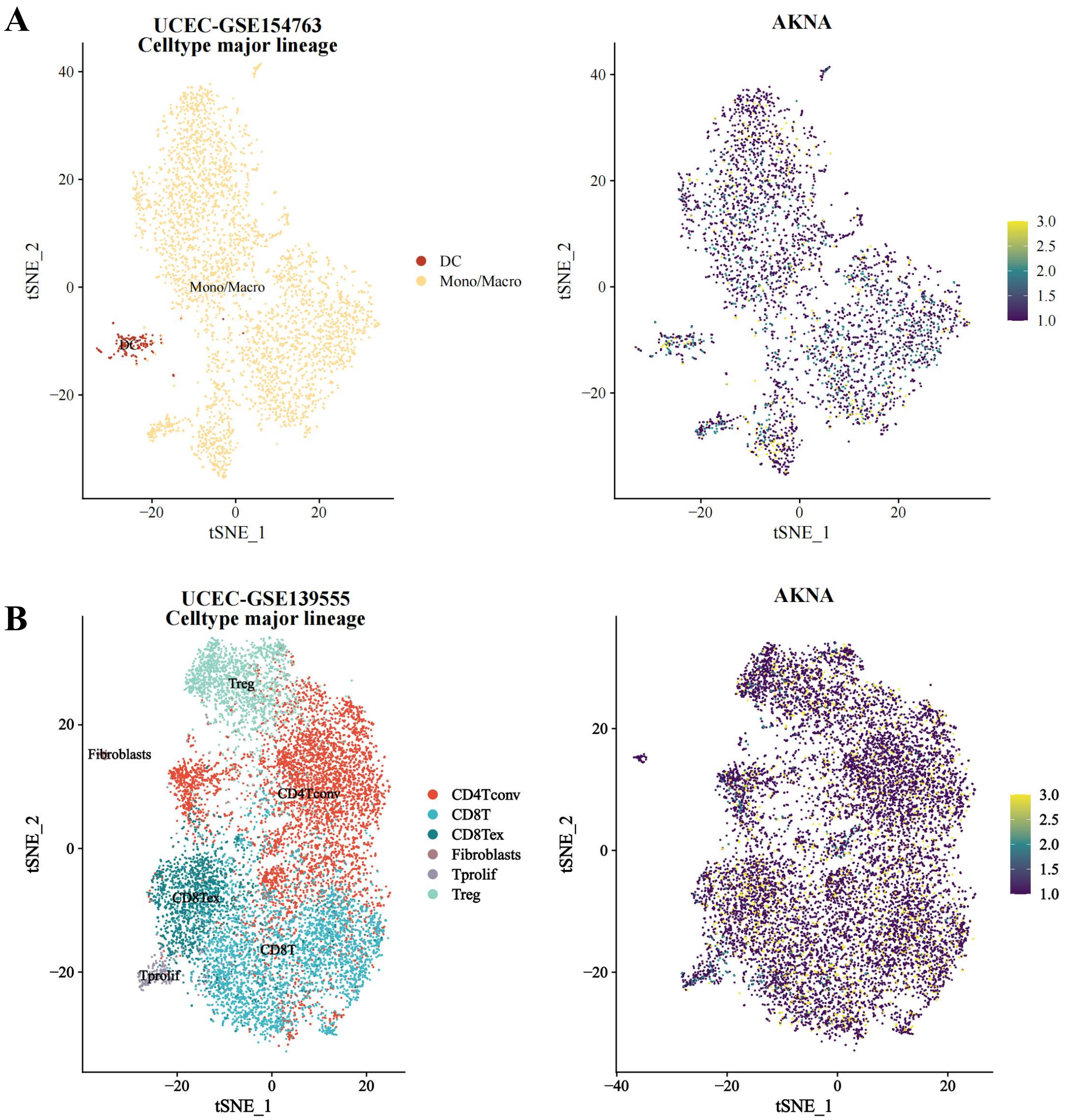

Supplementary Figure 3

Single-cell expression profiles of AKNA in EC. AKNA gene signature expression in the UCEC scRNA-seq datasets GSE154763 (A) and GSE139555 (B) retrieved from the TISCH database, demonstrating cell-type specific expression patterns across distinct cohorts.

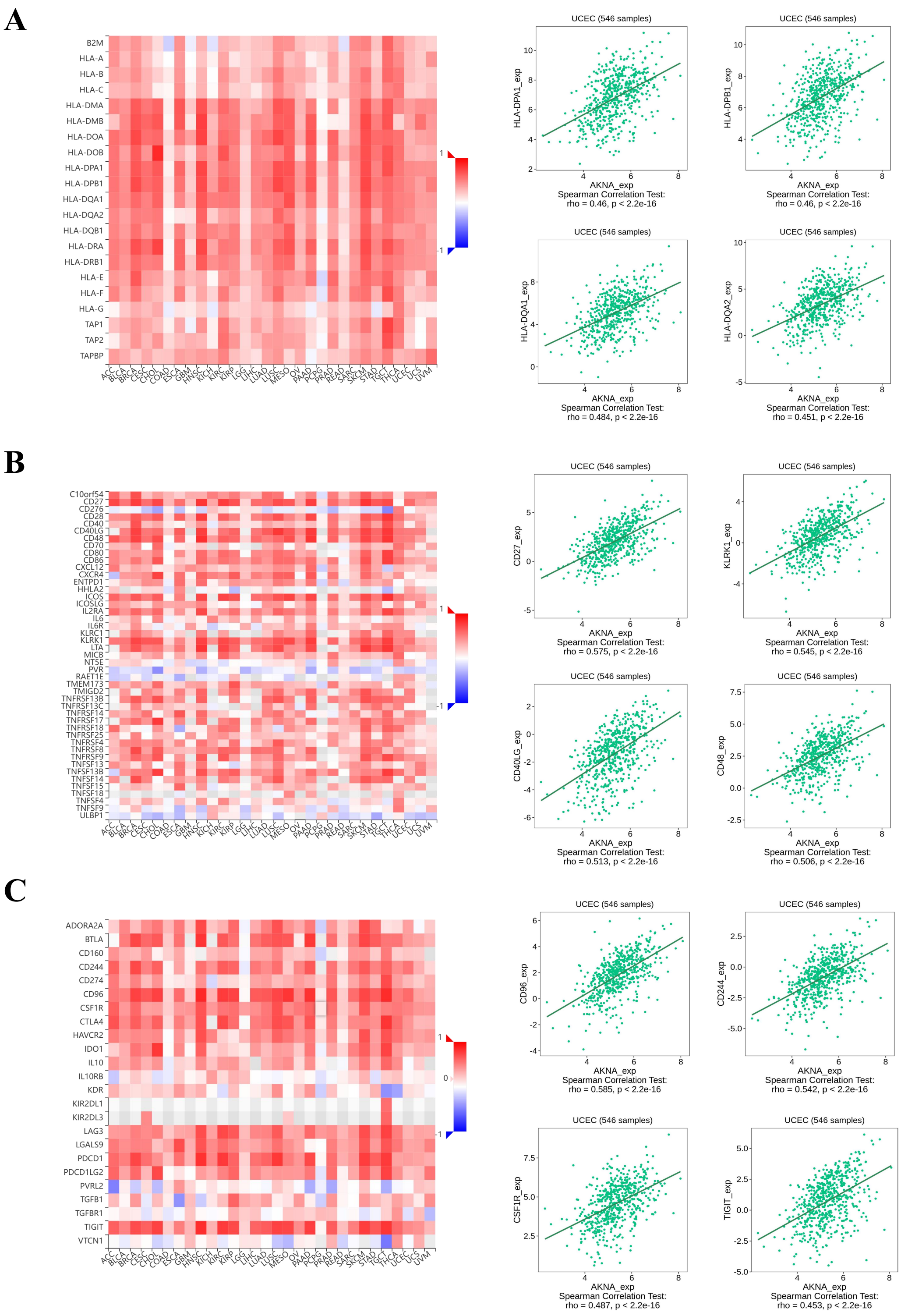

Supplementary Figure 4

Correlation between AKNA expression and immunomodulators in the tumor microenvironment. Left panels show pan-cancer Spearman correlation heatmaps; right panels show representative scatter plots in the TCGA-UCEC cohort. (A) Correlation between AKNA expression and major histocompatibility complex (MHC) molecules. (B) Correlation between AKNA expression and immunostimulators. (C) Correlation between AKNA expression and immunoinhibitors.

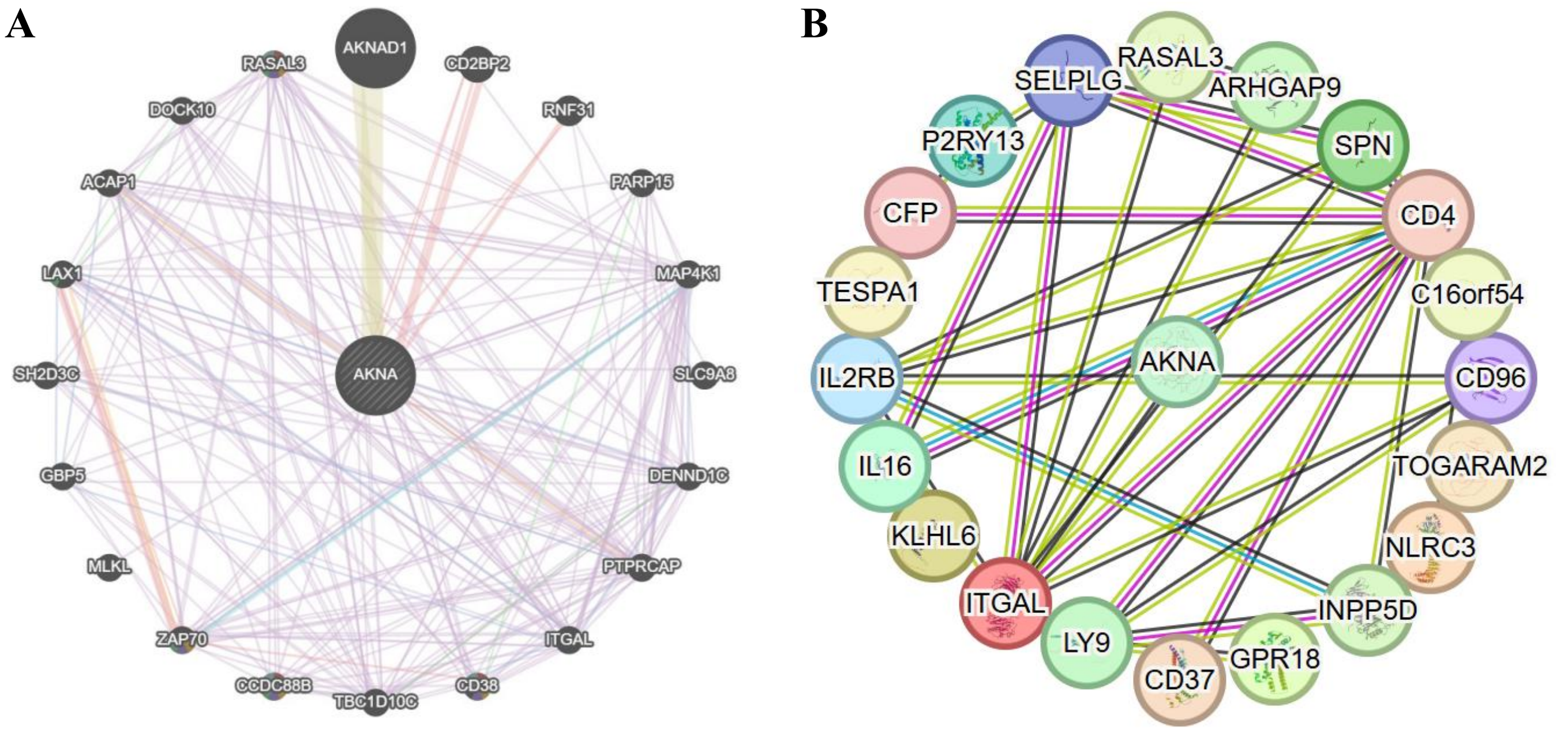

Supplementary Figure 5

Functional interaction and co-expression networks of AKNA. (A) AKNA co-expression and functional interaction network predicted via GeneMANIA. (B) Protein-protein interaction network of AKNA co-expressed genes in the TCGA-UCEC cohort, visualized using Metascape.

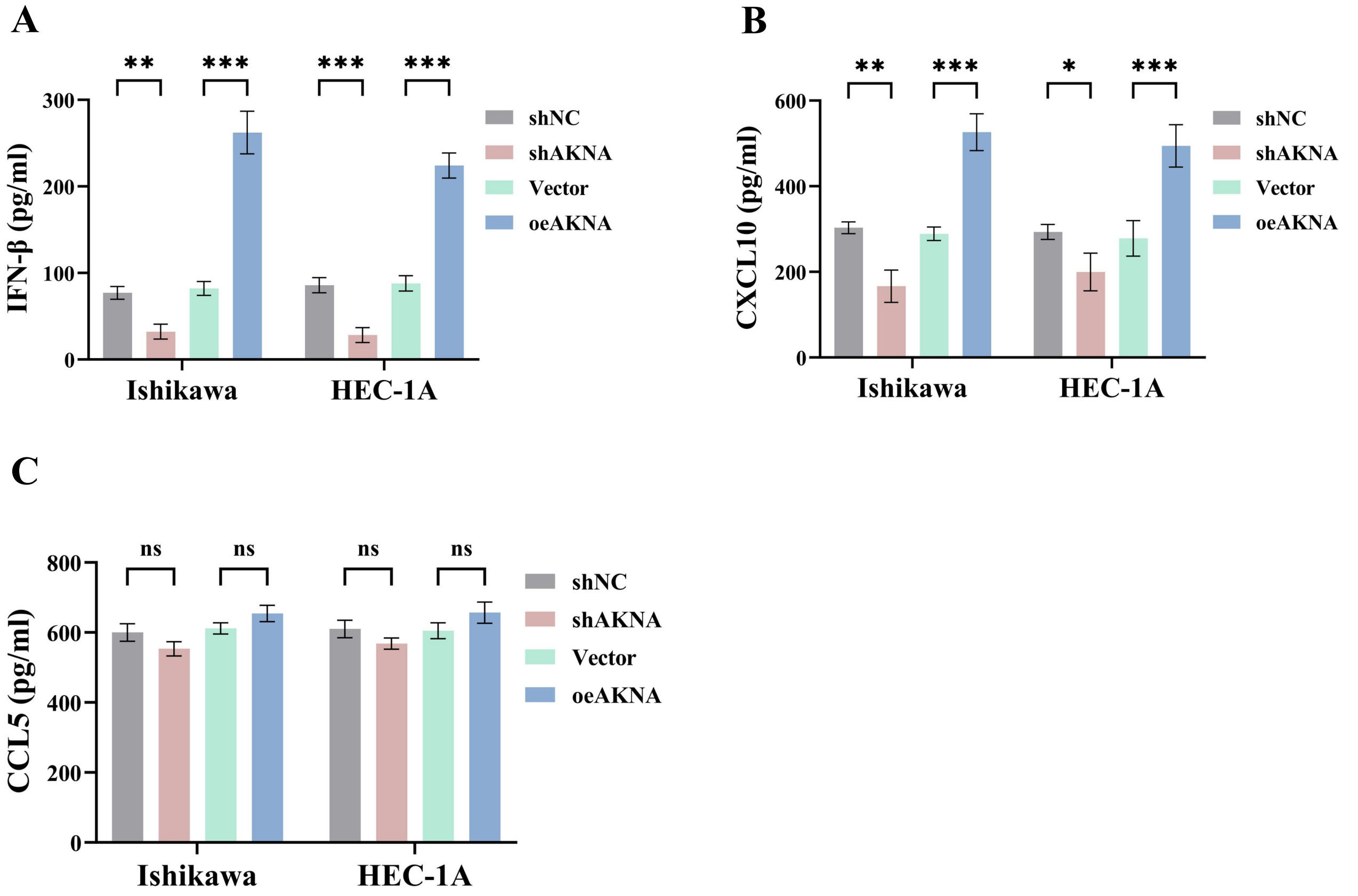

Supplementary Figure 6

AKNA modulates cytokine secretion in EC cells. ELISA quantification of cytokine secretion in conditioned medium from Ishikawa and HEC-1A cells transduced with lentiviruses for AKNA knockdown (shAKNA), overexpression (oeAKNA), or respective controls (shNC, Vector). **(A)** IFN-β secretion. **(B)** CXCL10 secretion. **(C)** CCL5 secretion. Data are presented as mean ± SD. Statistical significance was determined by one-way ANOVA. **P < 0.05, **P < 0.01, ***P < 0.001; ns, not significant.

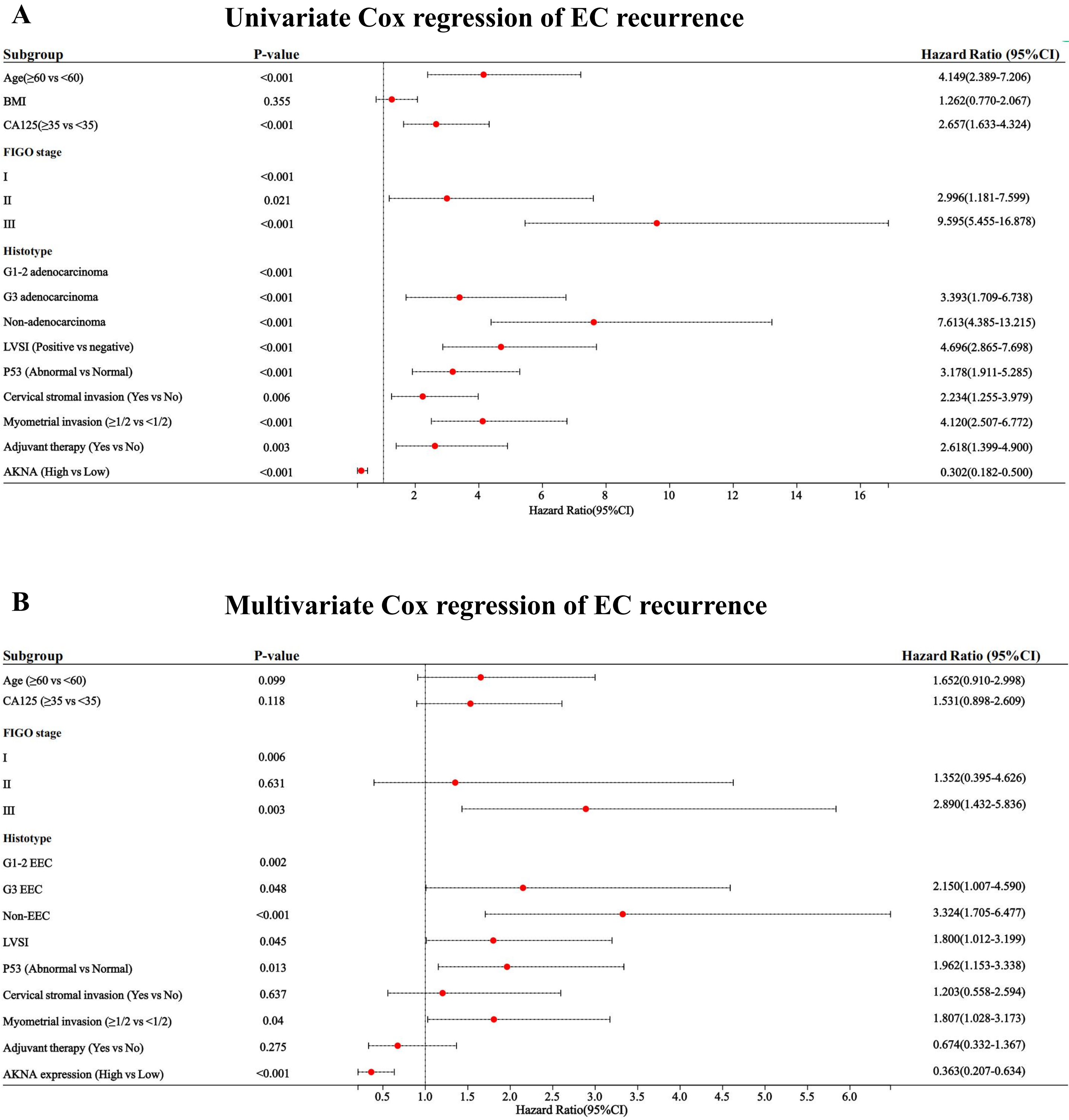

Supplementary Figure 7

Forest plots of Cox regression analyses for RFS in the training cohort. (A) Univariate Cox regression analysis of AKNA expression and clinicopathological factors associated with RFS. (B) Multivariate Cox regression analysis identifying independent prognostic factors for RFS in the training cohort.

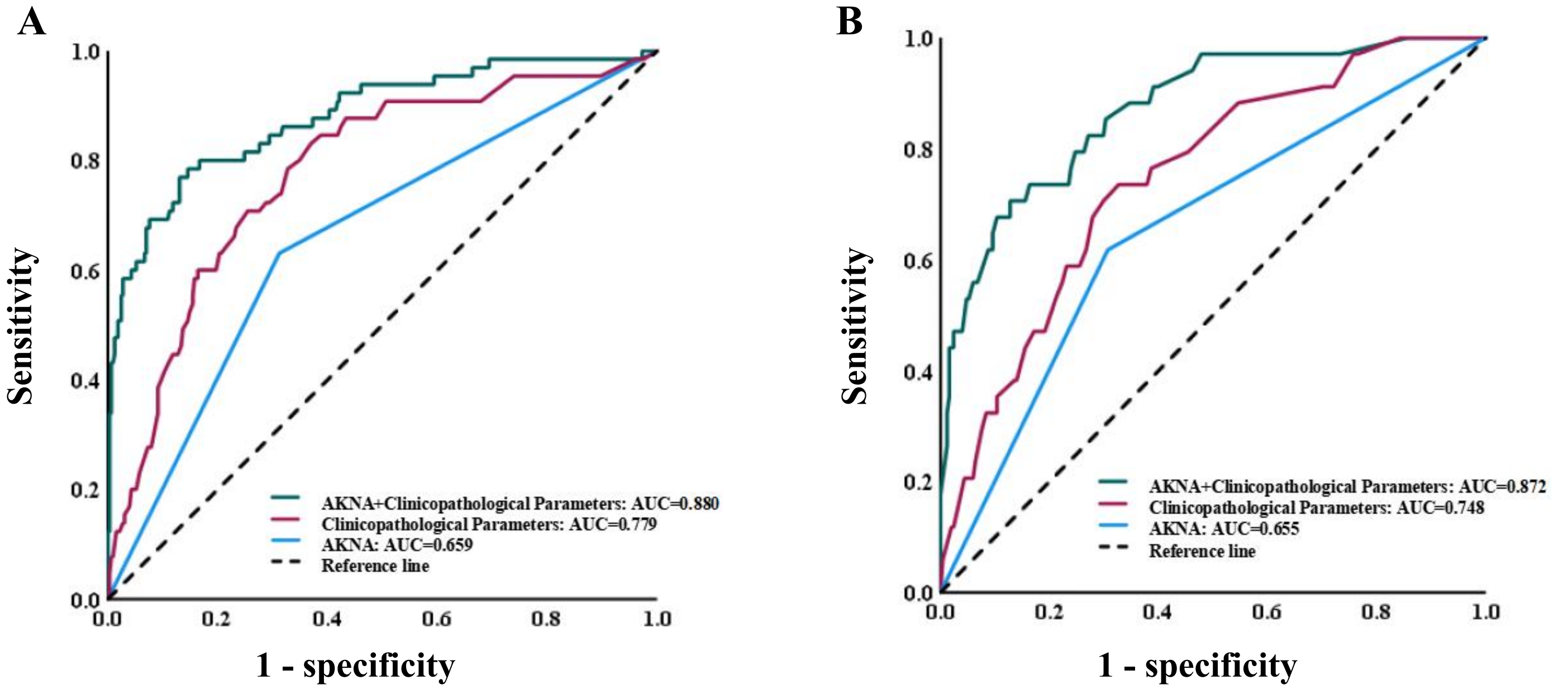

Supplementary Figure 8

Comparison of predictive performance between AKNA expression, clinicopathological factors, and the integrated model. (A, B) Time-dependent ROC curves showing the predictive accuracy (AUC) of AKNA alone, clinicopathological variables alone, and the combined prognostic model for RFS in the training cohort (A) and the validation cohort (B).

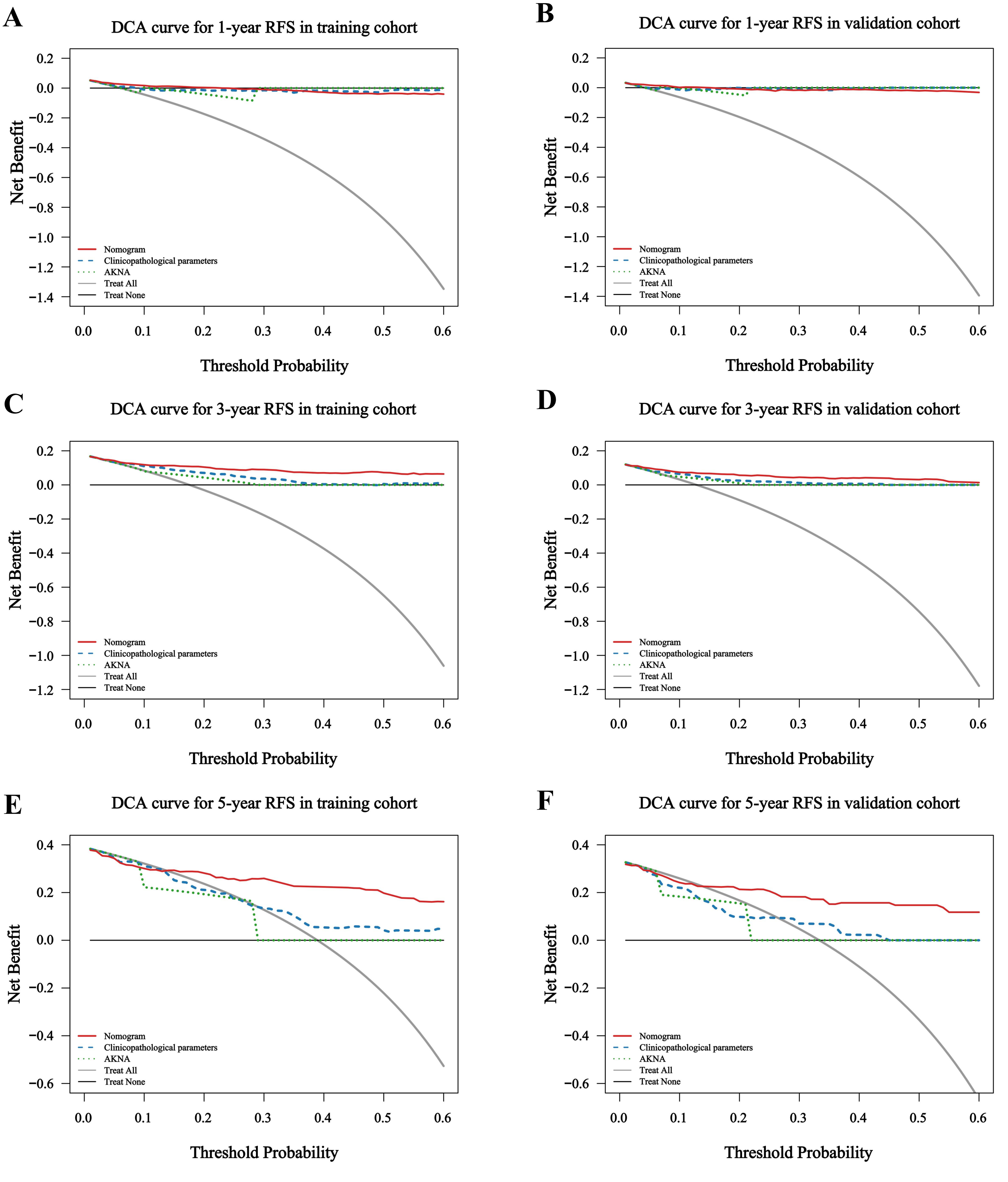

Supplementary Figure 9

Decision curve analysis (DCA) of the nomogram for predicting RFS. DCA curves for 1-year (A, B), 3-year (C, D), and 5-year (E, F) RFS in the training cohort and validation cohort. The solid red line represents the nomogram; the dashed blue line represents clinicopathological parameters alone; the dashed green line represents AKNA alone; the gray line represents the "treat-all" strategy; and the horizontal black line represents the "treat-none" strategy.

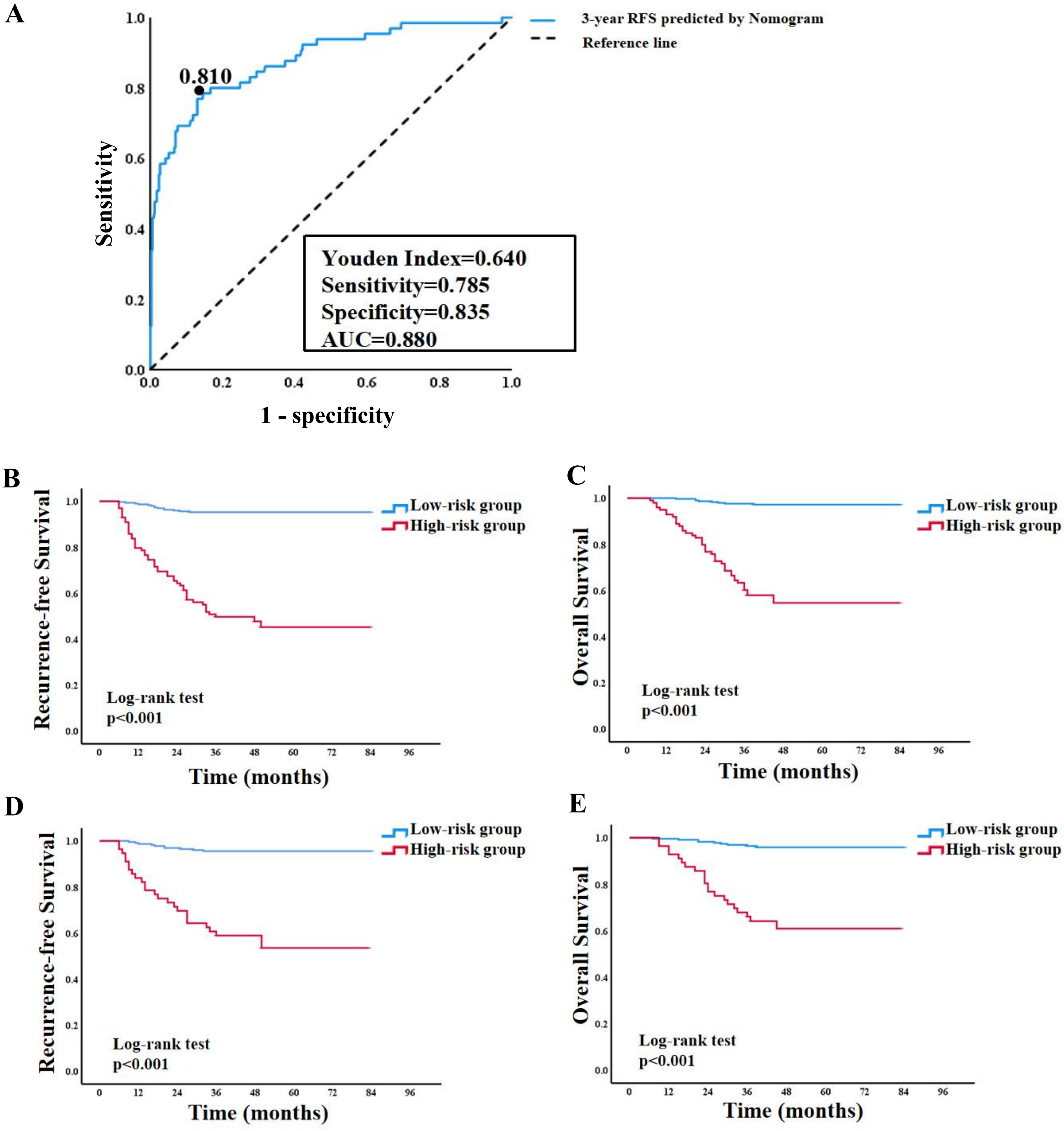

Supplementary Figure 10

Predictive performance and risk stratification efficacy of the integrated nomogram. (A) ROC curve analysis for 3-year RFS prediction in the training cohort. The AUC, sensitivity, and specificity are indicated based on the optimal risk threshold (0.810) determined by maximizing Youden's index. (B-E) Kaplan-Meier survival curves for RFS and OS in the training (B, C) and validation (D, E) cohorts based on nomogram-derived risk stratification.
