# Supplementary material for: AKNA serves as a prognostic biomarker and restrains endometrial cancer progression via STING activation and macrophage reprogramming
Source: Front Immunol. 2026 Oct 5;17:1905624. doi: 10.3389/fimmu.2026.1905624 (PMC13677899; doi:10.3389/fimmu.2026.1905624)
Supplement: Supplementary file 2 [file Table1.docx]

**Supplementary Table 1 Bioinformatics resources.**

| Databases | Abbreviation | URL |
| --- | --- | --- |
| Tumor Immune Estimation Resource | TIMER1.0 | https://cistrome.shinyapps.io/timer/ |
| Tumor-Immune System Interaction Database | TISIDB | http://cis.hku.hk/TISIDB/ |
| The University of ALabama at Birmingham CANcer data analysis Portal | UALCAN | https://ualcan.path.uab.edu/ |
| Kaplan-Meier Plotter | KM Plotter | https://kmplot.com/analysis/ |
| Gene Multiple Association Network Integration Algorithm | GeneMANIA | https://genemania.org/ |
| cBioPortal for Cancer Genomics | cBioPortal | https://www.cbioportal.org/ |
| Metascape Gene List Analysis Portal | Metascape | http://metascape.org/ |
| Wei Sheng Xin | WSX | https://www.bioinformatics.com.cn/ |
| Genomics of Drug Sensitivity in Cancer | GDSC | https://www.cancerrxgene.org/ |
| STRING Protein-Protein Interaction Networks | STRING | https://string-db.org/ |

**Supplementary Table 2 The Primers of RT-qPCR.**

| **Gene** | **Primer sequence for RT-qPCR** |
| --- | --- |
| AKNA | (Forward Primer)5′-GAGGTGAAGAGCAGATTGTC-3′ |
|  | (Reverse Primer)5′- GATTCTGAACTCAGGGACAG -3′ |
| STING | (Forward Primer)5′-CTACATTGGGTACTTGCGGTT-3′ |
|  | (Reverse Primer)5′-GCACCACTGAGCATGTTGTTATG-3′ |
| GAPDH | (Forward Primer)5′- GTCTCCTCTGACTTCAACAGCG-3′ |
|  | (Reverse Primer)5′- ACCACCCTGTTGCTGTAGCCAA-3′ |
|  | **Primer sequence for ChIP-PCR** |
| STING | (Forward Primer)5′-GACTCCACACACTCAGCCAAAG-3′ |
|  | (Reverse Primer)5′- TTCCTGCAAAGAGCCAAACC-3′ |

**Supplementary Table 3 Association between AKNA expression and clinicopathological characteristics in the UCEC cohort.**

| **Characteristic** | **Total patients**  **(*n* = 544)** | **High expression**  **(*n* = 273)** | **Low expression**  **(*n* = 271)** | ***P*** |
| --- | --- | --- | --- | --- |
| **Age, years**  <60  ≥60 | 181  363 | 96 (35.2%)  177 (64.8%) | 85 (31.4%)  186 (68.6%) | 0.347 |
| **FIGO stage**  I  II  III  IV | 339  52  123  30 | 184 (67.4%)  22 (8.1%)  58 (21.2%)  9 (3.3%) | 155 (57.0%)  30 (11.0%)  65 (23.9%)  21 (7.7%) | 0.031 |
| **Grade**  G1  G2  G3 High Grade | 99  122  312  11 | 67 (24.5%)  69 (25.3%)  133 (48.7%)  4 (1.5%) | 32 (11.8%)  53 (19.5%)  179 (65.8%)  7 (2.6%) | <0.001 |
| **Histological Subtypes**  EEC  Non-EEC | 407  137 | 215 (78.8%)  58 (21.2%) | 192 (70.8%)  79 (29.2%) | 0.034 |
| **TP53 Expression(n = 524)**  Wild  Mutant | 329  155 | 190 (69.6%)  74 (27.1%) | 139 (51.1%)  121 (44.5%) | <0.001 |
| **Adjuvant therapy**  No  Yes | 245  299 | 134 (49.1%)  139 (50.9%) | 111 (40.8%)  160 (59.2%) | 0.057 |

**Abbreviations:** FIGO, International Federation of Gynecology and Obstetrics; EEC, endometrioid endometrial carcinoma; EEC, endometrioid endometrial carcinoma

**Note:** TP53 status data were available for 524 of the 544 patients, as indicated in the table.

**Supplementary Table 4 Association between AKNA expression and clinicopathological characteristics of EC** **in the training cohort** **(*n* = 395).**

| **Characteristic** | **High expression**  **(*n* = 251)** | **Low expression (*n* = 144)** | ***P*** |
| --- | --- | --- | --- |
| **Age, years**  <60  ≥60 | 189 (75.30%)  62 (24.70%) | 94 (65.30%)  50 (34.70%) | 0.033 |
| **BMI, kg/m^2^**  Mean ± SD | 24.50 ± 3.91 | 24.52 ± 3.54 | 0.992 |
| **FIGO stage**  I  II  III | 184 (73.30%)  20 (8.00%)  47 (18.70%) | 87 (60.40%)  12 (8.30%)  45 (31.30%) | 0.015 |
| **LVSI**  Negative  Positive | 199 (79.30%)  52 (20.70%) | 93 (64.60%)  51 (35.40%) | 0.001 |
| **CA125, U/ml**  ≤ 35  > 35 | 190 (75.70%)  61 (24.30%) | 96 (66.70%)  48 (33.30%) | 0.053 |
| **Cervical stromal invasion**  Negative  Positive | 222 (88.40%)  29 (11.60%) | 123 (85.40%)  21 (14.60%) | 0.383 |
| **Myometrial invasion**  <1/2  ≥1/2 | 180 (71.70%)  71 (28.30%) | 96 (66.70%)  48 (33.30%) | 0.293 |
| **Pathological type**  G1-G2 EEC  G3 EEC  Non-EEC | 182 (72.50%)  32 (12.70%)  37 (14.70%) | 96 (66.70%)  22 (15.30%)  26 (18.10%) | 0.472 |
| **p53 expression**  Normal  Abnormal | 165 (65.70%)  86 (34.30%) | 78 (54.20%)  66 (45.80%) | 0.023 |
| **Adjuvant therapy**  No  Yes | 102 (40.60%)  149 (59.40%) | 38 (26.40%)  106 (73.60%) | 0.004 |

**Abbreviations:** BMI, body mass index; FIGO, International Federation of Gynecology and Obstetrics; LVSI, lymphovascular space invasion; EEC, endometrioid carcinoma; SD, standard deviation.

**Supplementary Table 5** **Clinicopathological Characteristics of Patients with EC in the Training and Validation Cohorts.**

| **Characteristic** | **Training Cohort**  **(*n* = 395)** | **Validation Cohort**  **(*n* = 285)** | ***P*** |
| --- | --- | --- | --- |
| **Age, years**  mean ± SD | 54.25 ± 9.51 | 53.88 ± 9.33 | 0.409 |
| **BMI, kg/m^2^**  mean ± SD | 24.51 ± 3.78 | 24.57 ± 3.94 | 0.561 |
| **FIGO stage**  I  II  III | 274 (69.4%)  30 (7.6%)  91 (23.0%) | 197 (69.1%)  21 (7.4%)  67 (23.5%) | 0.986 |
| **LVSI**  Negative  Positive | 292 (73.9%)  103 (26.1%) | 219 (76.8%)  66 (23.2%) | 0.385 |
| **CA125, U/ml**  ≤ 35  > 35 | 286 (72.4%)  109 (27.6%) | 214 (75.1%)  71 (24.9%) | 0.434 |
| **Cervical stromal invasion**  Negative  Positive | 345 (87.3%)  50 (12.7%) | 252 (88.4%)  33 (11.6%) | 0.671 |
| **Myometrial invasion**  <1/2  ≥1/2 | 276 (69.9%)  119 (30.1%) | 209 (73.3%)  76 (26.7%) | 0.325 |
| **Pathological type**  G1-G2 EEC  G3 EEC  Non-EEC | 278 (70.4%)  54 (13.7%)  63 (15.9%) | 207 (72.6%)  36 (12.6%)  42 (14.7%) | 0.814 |
| **P53 expression**  Normal  Abnormal | 243 (61.5%)  152 (38.5%) | 176 (61.8%)  109 (38.2%) | 0.950 |
| **Adjuvant therapy**  No  Yes | 140 (35.4%)  255 (64.6%) | 108 (37.9%)  177 (62.1%) | 0.512 |

**Abbreviations:** BMI, body mass index; FIGO, International Federation of Gynecology and Obstetrics; LVSI, lymphovascular space invasion; EEC, endometrioid carcinoma; SD, standard deviation.
